# The interplay of various sources of noise on reliability of species distribution models hinges on ecological specialisation

**DOI:** 10.1371/journal.pone.0187906

**Published:** 2017-11-13

**Authors:** Alaaeldin Soultan, Kamran Safi

**Affiliations:** 1 Max Planck Institute for Ornithology, Department of Migration and Immuno-ecology, Am Obstberg 1, Radolfzell, Germany; 2 University of Konstanz, Department of Biology, Universitätsstraße 10, Konstanz, Germany; Oregon State University, UNITED STATES

## Abstract

Digitized species occurrence data provide an unprecedented source of information for ecologists and conservationists. Species distribution model (SDM) has become a popular method to utilise these data for understanding the spatial and temporal distribution of species, and for modelling biodiversity patterns. Our objective is to study the impact of noise in species occurrence data (namely sample size and positional accuracy) on the performance and reliability of SDM, considering the multiplicative impact of SDM algorithms, species specialisation, and grid resolution. We created a set of four ‘virtual’ species characterized by different specialisation levels. For each of these species, we built the suitable habitat models using five algorithms at two grid resolutions, with varying sample sizes and different levels of positional accuracy. We assessed the performance and reliability of the SDM according to classic model evaluation metrics (Area Under the Curve and True Skill Statistic) and model agreement metrics (Overall Concordance Correlation Coefficient and geographic niche overlap) respectively. Our study revealed that species specialisation had by far the most dominant impact on the SDM. In contrast to previous studies, we found that for widespread species, low sample size and low positional accuracy were acceptable, and useful distribution ranges could be predicted with as few as 10 species occurrences. Range predictions for narrow-ranged species, however, were sensitive to sample size and positional accuracy, such that useful distribution ranges required at least 20 species occurrences. Against expectations, the MAXENT algorithm poorly predicted the distribution of specialist species at low sample size.

## Introduction

Understanding spatio-temporal distribution patterns of species is fundamental for ecology, conservation, biogeography, and many environmental studies. Species distribution model (SDM) allows for predictions of species distributions by quantifying relationships between species occurrence and associated environmental conditions [1–3]. SDM, which conceptually relies on ecological niche theory, is referred to by a number of alternative names, including: bio-climatic envelope model, habitat suitability model, ecological niche model and resource selection functions [2–4]. SDM is considered a powerful tool and is widely used to answer many ecological questions, such as the distribution patterns of invasive species [5,6], species responses to global change [7,8], identifying priority sites for conservation and suitable sites for reintroductions [9,10], and for conservation action plans [11]. One of the most widely used classes of SDM is a presence-background, which has been used in roughly 53% of SDM studies published between 2008 and 2014 [1]. This presence-background model compares the environmental conditions at the locations a species was recorded (henceforth referred to as the ‘species occurrence’) to other points (background or pseudo-absence), distributed throughout the environmental domain [1].

The increase in applications of presence-background SDM is partially based on the increased availability of freely accessible biodiversity data. These data provide an unprecedented amount of information for ecologists, conservationists, and biogeographers. However, these data come with analytical challenges, mainly due to the often-problematic noise they contain [12,13]. The noise in the species occurrence data originates from many sources, for instance: 1) low sample size due to limited data availability on species distribution [14–16], 2) low positional accuracy due to incomplete species record descriptions, digitization errors, or geo-referencing issues [13,17–19], 3) imperfect detection and misidentification of species due to less skilled or untrained observers [20–22], and 4) sampling bias resulting from non-systematic field surveys, biased data collection from relatively accessible areas, or using biased sampling efforts [23–25].

The impact of sampling bias on SDM has been extensively investigated, with several suggestions for bias correction [14,24–34]. The impact of sample size and positional accuracy on SDM, however, is still very much open for debate. Several studies have explored the effect of sample size [15,16,35–40], but despite a consensus that low sample size decreases accuracy, there is disagreement regarding the minimum number of species occurrences necessary to generate a useful distribution range.

Similarly, the positional accuracy of species occurrences has also been studied, however, there is no consensus regarding its impact on SDM. Some researchers suggest excluding low positional accuracy, while others argue that omission might negatively influence performance by reducing sample sizes [15,16,41]. Though some studies have found that SDM is generally insensitive to variation in positional accuracy level [12,42–45], there are others studies that disagree with such findings [17,18,46,47].

The impact of noisy data on SDM, specifically sample size and positional accuracy, can interact with other factors, such as grid resolution of the environmental data, model algorithm, and species specialisation. Previous studies have explored the additive effects of sample size or positional accuracy with some of these factors. For example, test combinations include the interaction between sample size, model algorithm, and species specialisation [15,35,39], or, sample size, model algorithm, and grid resolution of environmental data [38,42], or, positional accuracy and model algorithm [12,44], or finally positional accuracy and properties of environmental data [17,18,43,46]. To our knowledge, no previous study has comprehensively investigated the interactive effect of these factors on SDM’s performance and reliability in a single coherent framework.

The objective of this study is thus to understand the potentially interactive effects of sample size and positional accuracy of species occurrences, grid resolution of environmental data, species specialisation and niche modelling algorithms that typically affect empirical species distribution studies. We investigated the potential effects by modelling species distribution using a variety of modelling tools for four ‘virtual’ species characterized by different specialisation levels, each sampled with different intensities and different positional accuracies and modelled with different resolutions of environmental information. This allowed us to disentangle the effects of the various parameters without bias by assess performance against a known distribution range, which is near-impossible for real species. For each of our four species, we built suitable habitat models using five SDM algorithms and two grid resolutions each based on a varying number of species occurrences and with different levels of positional accuracy. We then investigated the subsequent variation in SDM performance and reliability. We assessed the outcome of the SDM based on classic model evaluation metrics (threshold-dependent and -independent), which are typically used in empirical studies because the actual distribution of the species in focus are unknown. However, unlike empirical species distribution studies, we “know” the real distributions, and can therefore directly measure the agreement between the SDM’s predictions and the “true” ranges; our actual goal for the modelling exercises.

## Materials and methods

**Fig 1:**
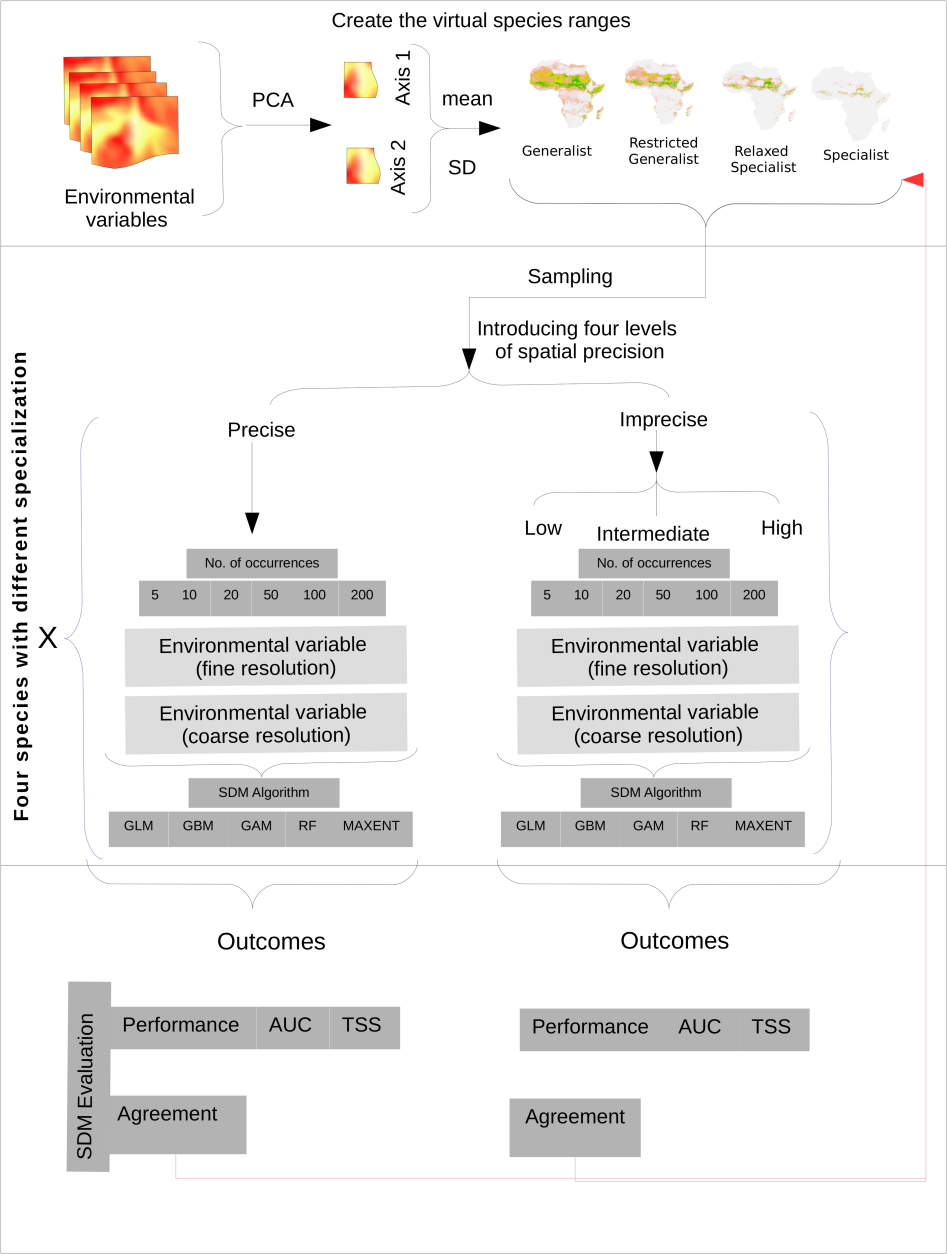
Flow diagram explaining the study design used to answer the study questions. The first step uses the first two axes of PCA and a Gaussian distribution function to create the four virtual species by adjusting the standard deviation (S.D.) value according to the species specialisation level. The second step shows the modelling process for these four species using five modelling algorithms with different sample sizes and different levels of positional accuracy (one precise and three increasingly imprecise levels) and two raster resolutions (high and low). The third step shows the evaluation procedure for model prediction based on the spatial agreement (reliability) and statistical performance (Area Under the Curve AUC and True Skill Statistics TSS).

Our analysis followed three steps: 1) creating ranges for four virtual species, 2) modelling species distribution ranges following the usual routine for each of the four species by sampling species occurrences from these ranges with different levels of positional accuracy, associating them with environmental information, and fitting species distribution models, and finally, 3) assessing SDM performance and reliability (Fig 1).

### Generating virtual species ranges

We based our analyses on species with known distribution ranges for unbiased model quality evaluation. To this end, we created ranges for the virtual species (henceforth referred to as the ‘“true” range’) using four environmental variables: 1) annual mean temperature, 2) altitude, 3) precipitation seasonality, and 4) annual mean evapo-transpiration. These environmental variables are widely considered to have a direct influence on the eco-physiology and niche of many species [16,48–51]. We downloaded these environmental variables at two resolutions, 2.5 and 10 arc-min, (the source of these variables in Table A in S1 Appendix). Variables only available only in higher resolution were downscaled to 2.5 and 10 arc-min resolutions using bilinear interpolation. We conducted a Principal Component Analysis (PCA) on these four environmental variables, and in order to avoid unrealistic distribution ranges due to species response to each of the four variables, we chose the first two PCs summarizing the environmental variability across the study area [52,53]. This is an objective and realistic approach and ensures that the ranges of the virtual species were delineated based on realistic environmental variables [27,52,53]. We defined the environmental range inhabited by the species based on the mean ± standard deviation (S.D.) for each of the first two axes of the PCA using a Gaussian distribution function. [16,27,54–56]. We then set the mean value (optimum of the environmental range) of the first two axes of the PCA at (0,0) for all species, and determined the degree of specialisation by adjusting the S.D. values of the first two axes of the PCA according to the species specialisation. The generalist species were characterized by low specialisation covering 80% (S.D. 0.8) of the environmental range. The restricted generalist, relaxed specialist and specialist species had ranges covering 60%, 40% and 20% of the environmental range respectively (Fig A in S1 Appendix).

The overall environmental suitability of each virtual species was computed by using the multiplicative approach, multiplying the output of the Gaussian distribution function of each of the first two PCs. We considered this approach to be more realistic, since it accurately represents the interaction between the environmental variables. For example, if one environmental variable was very unfavourable at a given location, the species’ probability of occurrence will be low overall, despite the other variables being close to the species’ optimum [27,51,54,57,58]. Finally, the defined environmental ranges were projected onto the world at the African continental scale at two different grid resolutions: 10 arc-min, representing the low resolution (spatial unit size ≈ 400 km2), and 2.5 arc-min, representing the high resolution (spatial unit size ≈ 25 km2) (Fig A in S1 Appendix). We used the “virtualspecies” R package to generate these four virtual species [52].

### Sampling species occurrences and introducing different positional accuracy levels

The threshold approach is widely used in simulation studies, where a fixed threshold is being selected to convert the probability of occurrence into a binary presence–absence map [27,52,59–61]. This threshold is arbitrarily selected since there are no objectively justifiable threshold values based on the data and/or on the validation values (e.g. sensitivity and specificity) [27]. However, using the threshold approach is problematic and has been criticized for numerous reasons: 1) it maximises the omission and commission errors rate in species occurrences, 2) it alters the predefined species-environment relationship and, 3) it is inappropriate for many of the regression models that rely on logistic functions, which in turn might provide misleading results [52,59]. To mitigate these issues, we used the threshold and probability based approaches together. First, we selected arbitrarily a threshold of 0.2 to convert the probability map into a binary, presence-absence map. Next, from the corresponding probability map, we used the values of the probabilities of occurrence in each pixel as the success rate for one sample of the binomial distribution (i.e. a pixel with a probability of 0.8 has an 80% chance of being occupied by species) [4,27,62]. In practice, for each pixel in the presence area in the binary map we generated a random value (r) on the interval [0,1], where a pixel was considered “present” if its r value was greater than its probability of occurrence. Similarly, the absence data was obtained by drawing pixels from the absence area in the binary map, where a pixel was considered “absent” if its r value was less than its probability of occurrence. This resulted in pixels with higher suitability to be more likely to be identified as “present” and pixels with lower suitability more likely identified as “absent” [4,18,27,51,59,62,63]. Thus, we tried to minimize the omission and commission error in species occurrences.

Next, we shifted the sampled species occurrences in a random direction to introduce four levels of positional accuracy [12,17,43,64]. To this end, we created four buffer areas around each occurrence and randomly sampled occurrence outside the buffer area to represent four levels of positional accuracy: 1) precise, where the buffer area size was zero to represent no change in the positional accuracy, 2) low imprecision, where the buffer area size was equal to one pixel, corresponding to ≈ 6 km at the high resolution and ≈ 20 km at the low resolution, 3) intermediate imprecision, where the buffer size was equal to two pixels, 4) high imprecision, where the buffer size was equal to three pixels.

### Modelling framework

#### Environmental variables

We followed the standard SDM’s routines for selecting the predictors, where typically the causal relationship between occurrence and environmental conditions is unknown. We do acknowledge that a poor choice of predictors is another common source of uncertainty in SDM’s studies. However, our objective here is to mimic the empirical SDM’s routines and assess how far predictions are from the reality under realistic conditions irrespective of the appropriateness of choosing the right set of predictors (which most empirical studies also are unaware of to begin with). We therefore selected 19 climatic predictors, two topographic predictors, five vegetation predictors, and one aridity variable to model distribution ranges at our two grid resolutions. We reduced the number of predictors by calculating the Variance Inflation Factor (VIF); a measure for collinearity. We removed collinearity by eliminating predictors with VIF scores greater than 10 [65], using the “vifstep” function in the “usdm” R package [66]. Finally, 15 predictors remained to build the SDM (Table A in S1 Appendix), which was sufficient to avoid model over-fitting and develop an accurate SDM [67]. We rescaled all predictors to the two different grid resolutions we used in our study (2.5 and 10 arc-min) using bilinear interpolation [68].

#### SDM algorithms

We modelled the ranges of each species using five commonly used algorithms that are either regression-based or machine learning-based approaches. We used two algorithms from the regression-based approaches: the Generalized Linear Model (GLM) [69,70], a widely used linear regression method, and the Generalized Additive Model (GAM) [70,71], a closely related method allowing for non-linear relationships. We used three implementations of machine learning-based approaches: Generalized Boosted Model (GBM) [72], Random Forest (RF) [73] and Maximum Entropy Modelling (MAXENT) [68,74], which characterize the environmental space directly from calibration data [67]. We fitted the models using the “Biomod2” R package [75].

#### Modelling procedure

To determine the acceptable minimum number of species occurrences, we calibrated the SDM for each species at the two grid resolutions using the five algorithms with different sample sizes (5, 10, 20, 50, 100 and 200 occurrences) with five-fold cross-validation and five replicates, where each replicate used a different background set, i.e., each model ran 25 times. We acknowledge that using species-specific model parameter tuning is recommended [76], however, to avoid an overwhelming complexity of the study outcome and also for the benefit of a better comparison between the algorithms, we decided to keep the default settings of the respective SDM algorithms (Table B in S1 Appendix). To determine the acceptable level of the positional accuracy of species occurrences, we repeated the procedure as described using imprecise occurrences (low, intermediate, and high) to compare with precise occurrences (Fig 1).

### Model evaluations

Model evaluation is a crucial step in model selection and assessing the accuracy of the prediction [77]. In general, model accuracy is measured mainly through evaluation and agreement metrics [78,79]. Evaluation metrics are widely used to measure model performance through assessing the ability of a model to distinguish between presence and absence locations correctly [78,79]. Agreement metrics, however, measure prediction reliability by assessing the spatial agreement between the “true” and predicted ranges taking into account the probability values of pixels. In other words, reliability can be used to inform how far the predicted ranges are from the truth or “reality” [78,79]. Using different evaluation metrics is strongly preferred when true absence data are unavailable, and also when the goal is to model potential distribution ranges rather than realized ranges [80]. Therefore, we calculated the area under the curve (AUC) of the receiver operating characteristic (ROC), as well as the True Skill Statistic (TSS) to evaluate the predictive performance of the models. The AUC value (a threshold-independent evaluation metric) ranges from 0 to 1, with values below 0.5 indicating performance no better than random, whereas a value of 1 indicates perfect performance [77]. TSS value (a threshold-dependent evaluation metric) varies from -1 to 1, where a value of 1 indicates perfect model performance, and a value lower than or equal to zero indicates a model performance no better than random [81]. In this study, we considered the models with either median AUC value ≥ 0.7 or median TSS value ≥ 0.4 as good models with usefully predictive distribution ranges (successfully able to discriminate the suitable from unsuitable areas) [43,82–85]. We used the “Biomod2” R package [75] to calculate the evaluation metrics (AUC and TSS) for each SDM internally as usually done in empirical studies (henceforth referred to as ‘standard AUC’ and ‘standard TSS’). Additionally, we evaluated the SDM by calculating AUC and TSS using independent data (presence and “true” absences) sampled from the true ranges (henceforth referred to as “independent AUC” and “independent TSS”). We calculated these independent metrics using the “accuracy” function in the “SDMTools” R package [86]. We compared the differences between the independent evaluation and 25 model evaluation metrics using one-sample *Wilcoxon test* using the “stats” R package [87]. To test whether the grid resolutions of the environmental predictors influenced model performance, we assessed the differences between the standard evaluation metric (standard AUC and standard TSS) values at the high and low grid resolutions for all models using two-sample non-parametric *Wilcoxon test*.

We assessed the interaction of spatial resolution, SDM algorithm, positional accuracy, sample size, and species specialisation on SDM’s performance using generalised linear models. We fitted two models, first fitting the exponentially transformed AUC as a function of spatial resolution, the SDM algorithm, positional accuracy, sample size, and species specialisation. In a second model, we additionally included the two-way interaction of these factors. We used the Akaike Information Criterion (AIC) to select the most parsimonious model favouring a low AIC value [88].

#### Measuring spatial agreement

We measured relative agreement between “true” and modelled ranges by calculating their geographical niche overlap. We calculated *Schoener's D* index [89] using the “nicheOverlap” function in the “dismo” R package [90]. The niche overlap value varies between 0 and 1, where the value of 0 indicates no overlap and value of 1 indicates complete overlap [91,92]. Additionally, we measured the absolute agreement between the “true” and modelled ranges through a pixel wise comparison using the Overall Concordance Correlation Coefficient (OCCC), a measure of agreement between two continuous datasets which were generated using two different approaches [93]. We computed the OCCC using the “epiR” R package [94]. The OCCC value varies between 0 and 1, with 0 representing 100% disagreement and 1 represents 100% agreement between the true and predicted ranges (See S2 Appendix for details).

## Results

### Minimum sample size of species occurrences required for SDM prediction

**Fig 2:**
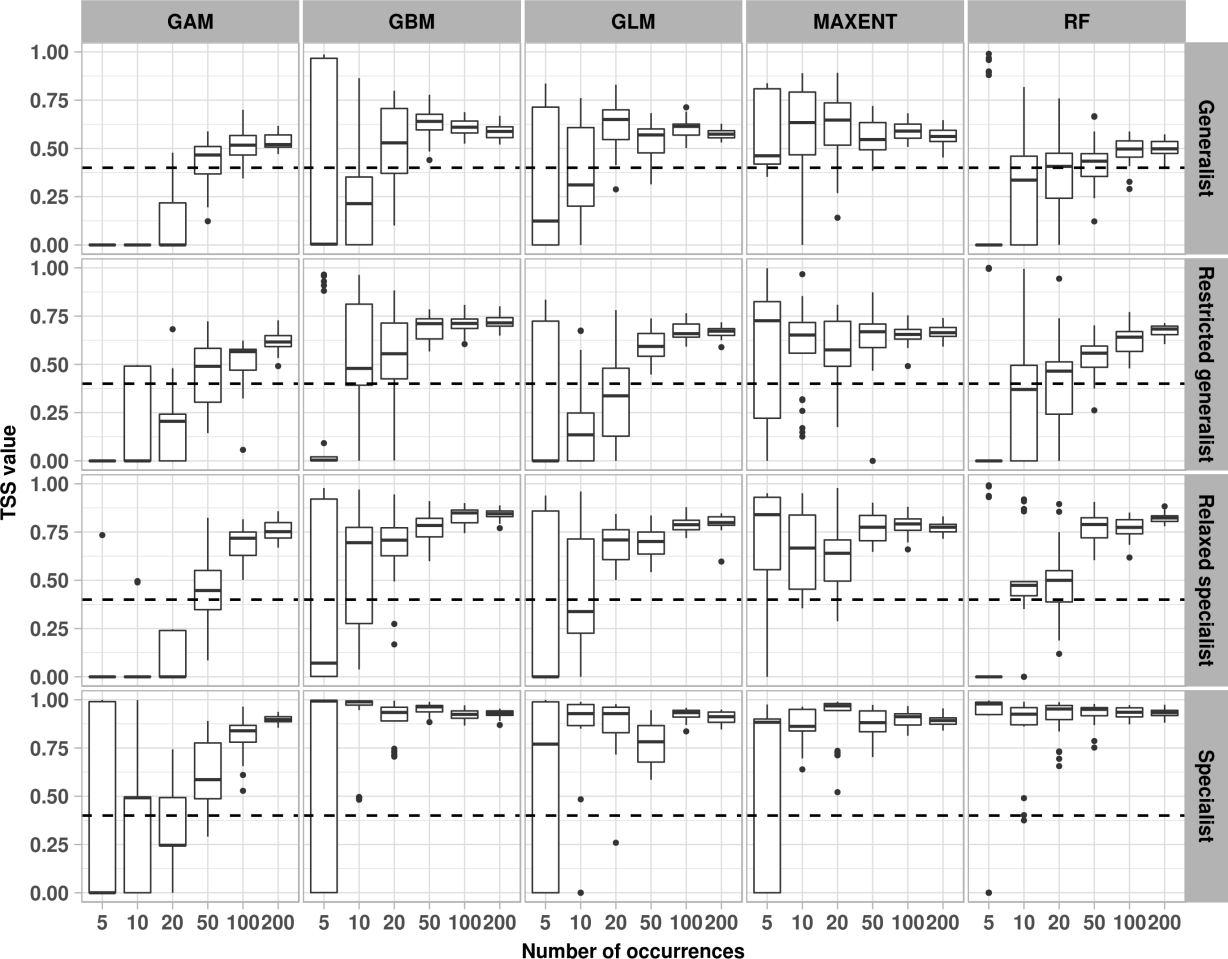
The inter-quantile range of the standard True Skill Statistic (TSS) at high grid resolution. This plot shows the variation in model performance for four species (row-wise) with increasing the sample size (x axis) using five different SDM algorithms (column-wise). The dashed line represents the threshold line, where median values above this line indicate good performance.

Our results revealed inconsistencies between the evaluation and agreement metrics regarding the minimum sample size of species occurrences required for SDM. The evaluation metrics showed that MAXENT was the only algorithm that successfully modelled the distribution range with five species occurrences regardless of the species specialisation. In contrast, GAM failed to successfully model the distribution ranges with fewer than 50 species occurrences. Though GLM, GBM, and RF required minimum 20 species occurrences to successfully model the distribution ranges for generalist and restricted generalist species, only five species occurrences were required for successful modelling of relaxed specialist and specialist species (Fig 2 and Figs B–D in S1 Appendix).

**Fig 3:**
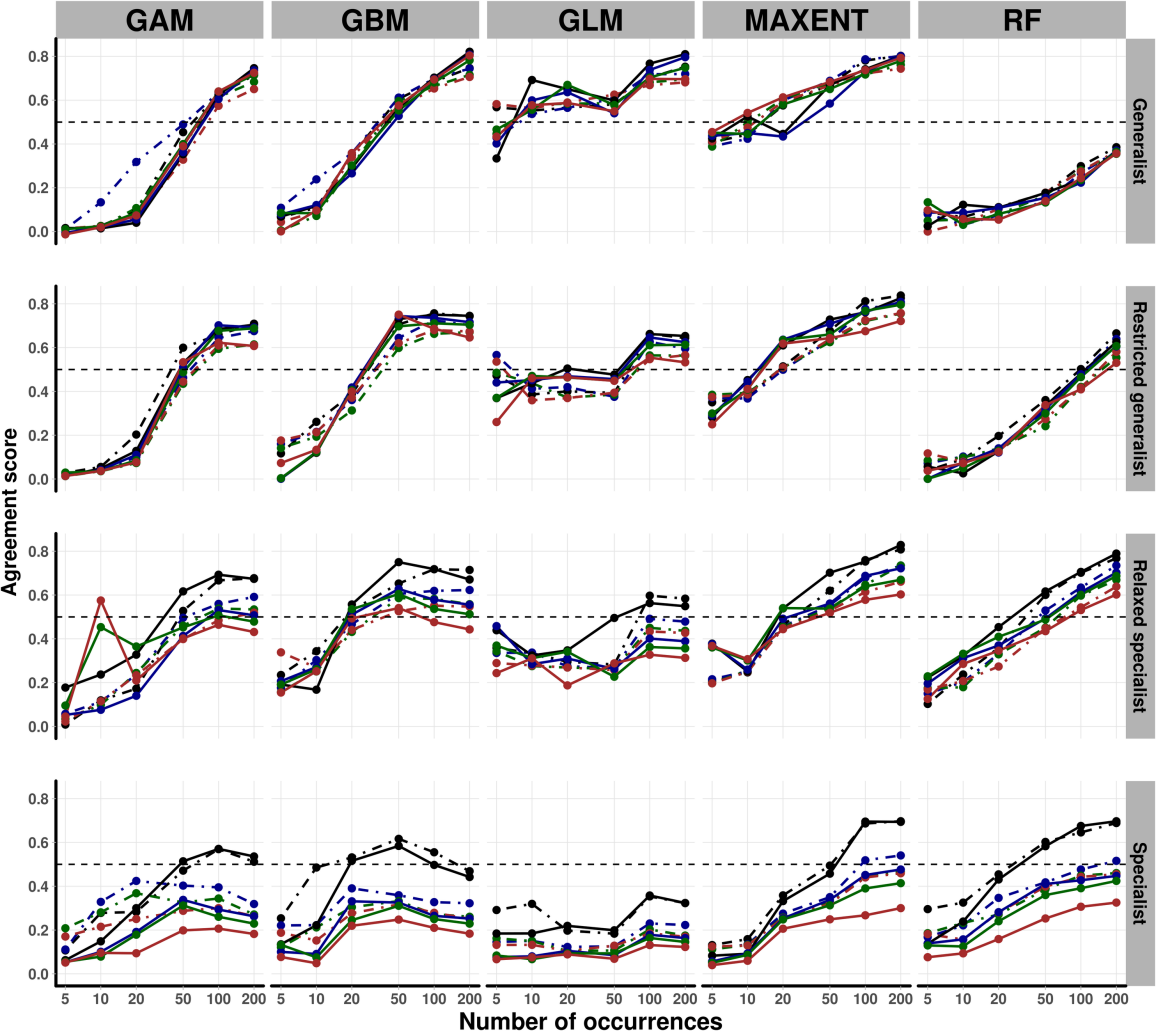
The agreement index. The spatial agreement between the predicted ranges with precise species occurrences and the predicted ranges with imprecise species occurrences for four different species at high and low grid resolutions–according to Overall Concordance Correlation Coefficient (OCCC) index. The y-axis is scaled from 0 to 1, where 0 means no agreement and 1 is 100% agreement. Solid lines represent low grid resolution and dashed lines represent high resolution. Line colour denotes the precision levels of the species occurrences, where the black line denotes precise species occurrence, the blue line denotes low imprecise, the green line denotes intermediate imprecise, and the red line denotes highly imprecise species occurrences.

In contrast, the agreement metrics showed that both MAXENT and GLM required a minimum of 10 species occurrences to model the ranges of generalist and restricted generalist species to ≈ 50% agreement with the “true” ranges. However, for specialist and relaxed specialist species, MAXENT required 50 occurrences to achieve ≈ 40% agreement with the “true” ranges, and GLM failed to achieve a similar agreement, even with 200 occurrences. Both GBM and RF required a minimum of 20 occurrences to achieve ≈ 45% agreement with the “true” ranges, whereas, for generalist and restricted generalist species RF could not achieve 40% agreement even with 100 occurrences (Fig 3 and Figs E–L, and P in S1 Appendix).

These findings are indicative of the presence of the interactive effect of species specialisation and the SDM algorithms on the number of species occurrence required for SDM, where, the number of species occurrences required for a good SDM varied according to the species specialisation and the type of algorithm used (Figs 2 and 3 and Figs B–D, O and P in S1 Appendix).

Our results suggested statistically significant differences between the standard and independent evaluation metrics according to the *Wilcoxon test*, however, the magnitude of these differences were relatively small, implying that these two metrics are practically similar (Tables C–F and Figs M and N in S1 Appendix).

Our results also revealed that grid resolution had no considerable effect on SDM compared to species specialisation and model algorithm (Fig 3 and Figs O and P in S1 Appendix). Although there was a statistical difference between the high and low grid resolutions, this effect size was relatively small (Tables G and H in S1 Appendix). Moreover, the difference was not consistent: in some cases, models at high grid resolution performed better than those based on low grid resolution, whilst in other cases models using low grid resolution performed better.

### Impact of positional accuracy of species occurrences on performance of SDM

**Fig 4:**
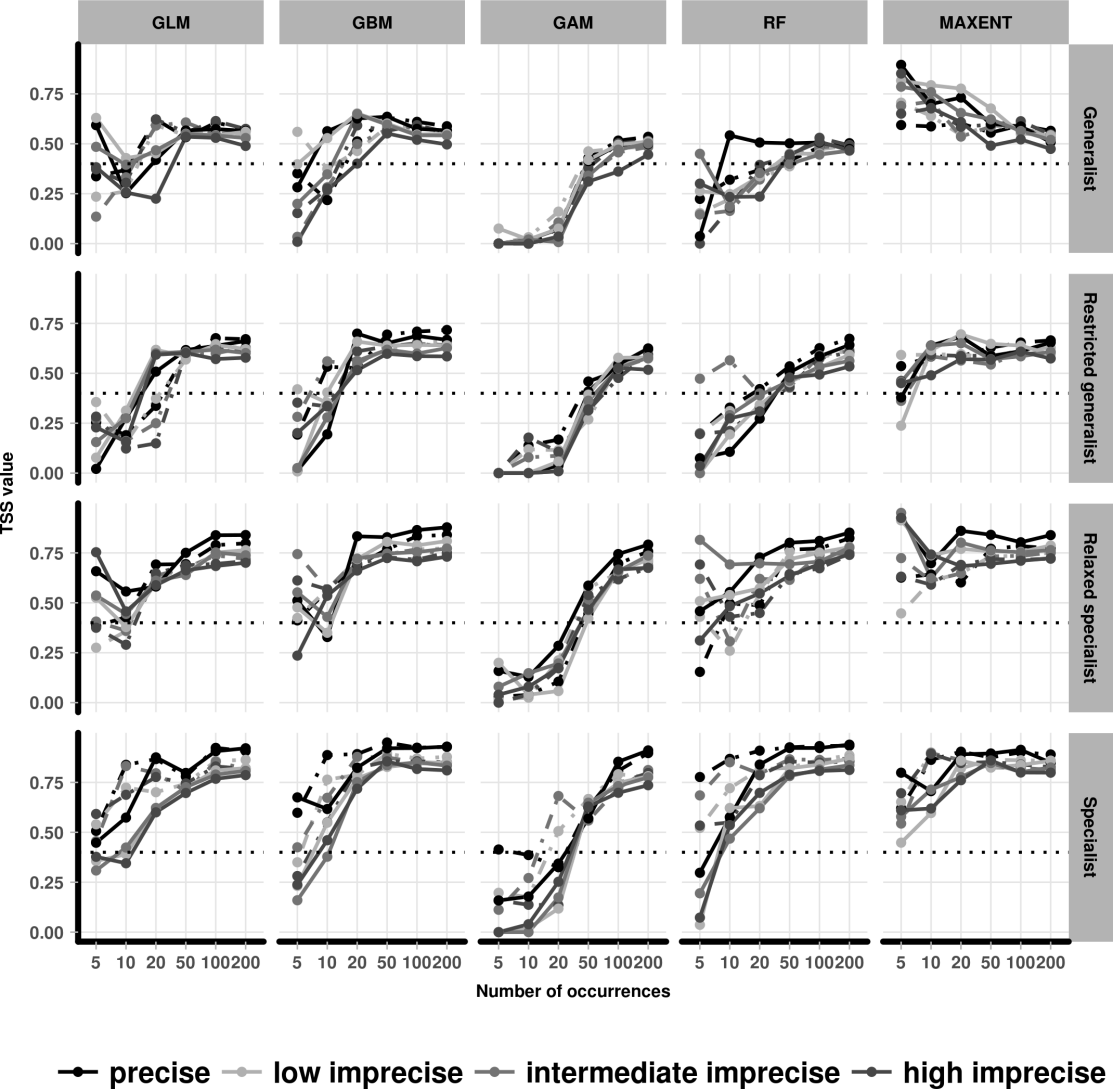
True Skill Statistic (TSS) for the models fitted with precise and imprecise occurrences. The variation between the performance of SDMs fitted with precise and imprecise species occurrences with different sample size (x axis) using five different SDM algorithms (column-wise) for four species with difference specialisation levels (row-wise). Line colour represents the precision levels of the species occurrences. Solid lines represent low grid resolution, and dashed lines represent high resolution. Dotted line is the threshold value below which poor model performance is indicated.

The models based on precise species occurrences tended to perform slightly better than those based on imprecise occurrences (low, intermediate and high). However, in some instances, when low sample sizes were used the models based on the imprecise species occurrences outperformed those based on precise occurrences. Models fitted with imprecise species occurrences had a clear tendency to reduce SDM performance in relaxed specialist and specialist species, which disappeared with sample sizes above 20 occurrences (Fig 4 and Fig O in S1 Appendix).

The result of the linear models indicates a significant influence of the interaction between spatial resolution, SDM algorithm, positional accuracy, sample size, and species specialisation on the SDM’s performance (delta AIC >5000). Species specialization and sample size were the most influential variables (in terms of the effect size), whereas spatial resolution and positional accuracy were the least influential variables (Table 1). The full set of the explanatory variables modelled is presented in supplementary file (Table I in S1 Appendix).

**Table 1:** Result of the linear model analysis investigating determinants of area under the receiver operating characteristic curve (AUC) values. Exponentially transformed AUC values were modelled as a function of spatial resolution, SDM algorithm, positional accuracy, sample size, and species specialisation. Akaike Information Criterion (AIC) showed that the full model with interaction was the less parsimonious model with AIC = -66657.46.

	Degree of freedom	Delta AIC	Adjusted R-squared
Full model with interaction	961	0	0.578
Full model without interaction	18	5002.8	0.458
- Spatial resolution	1	5014.46	0.458
- Positional accuracy	3	5222.46	0.371
- Algorithm	4	8542.46	0.453
- Species specialisation	3	10101.46	0.221
- Sample size	5	13622.46	0.328

### Impact of positional accuracy of species occurrences on reliability of SDM

Both niche overlap and OCCC indicated a strong spatial agreement between ranges modelled with precise and imprecise species occurrence data for generalist and restricted generalist species. This agreement weakened with decreasing positional accuracy, and increasing specialisation. Moreover, this agreement also weakened with increasing numbers of imprecise species occurrences, and differences were more pronounced at low grid resolution (Fig 3 and Fig P in S1 Appendix). These findings were consistent across the five algorithms. In general, our results suggest an interaction between sample size and positional accuracy, SDM algorithms, species specialisation, and grid resolutions on the reliability of SDM.

## Discussion

**Fig 5:**
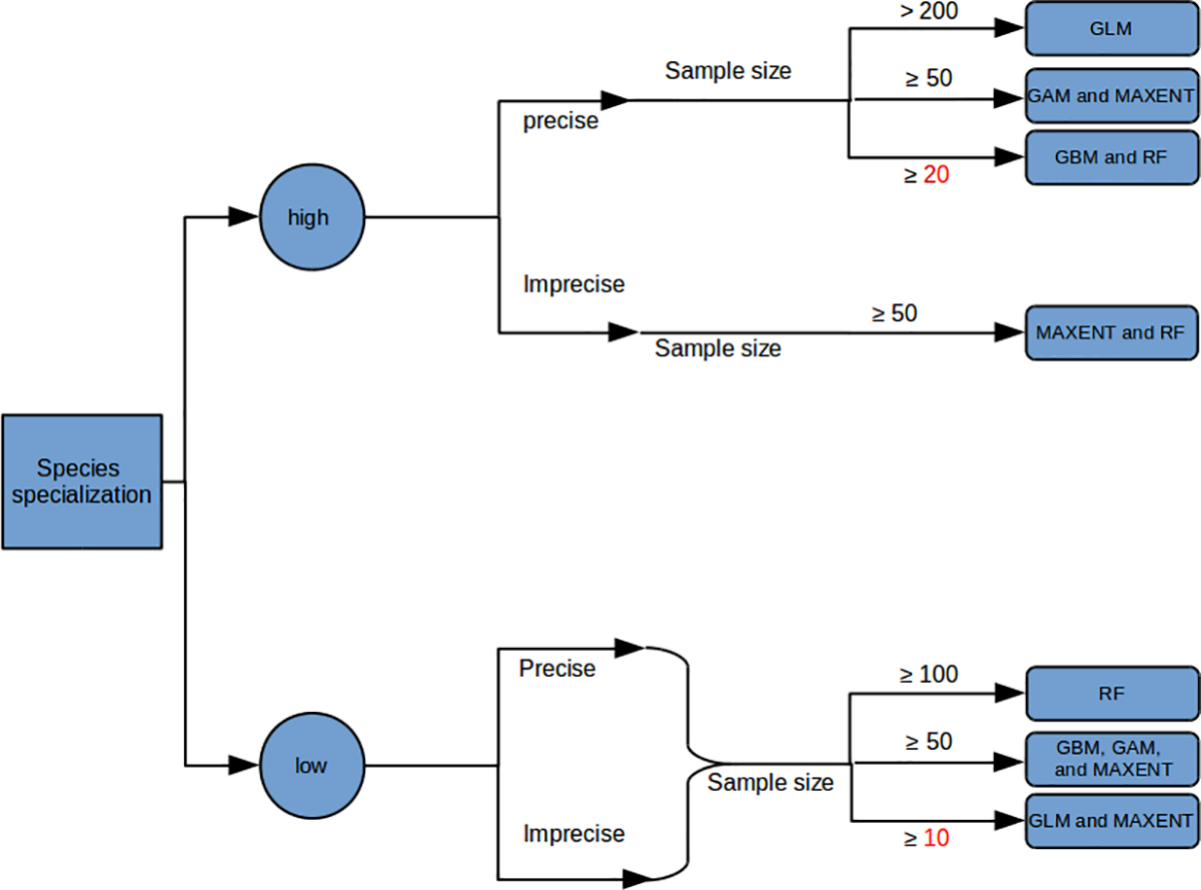
Framework demonstrates the factors that need to be considered depending on the characteristics of species specialisation. The number represents the minimum sample size of occurrences that is needed to model the SDM according to the positional accuracy of species occurrences and algorithm type.

Our comprehensive analysis uncovered how the sample size and positional accuracy of species occurrences, model algorithms, grid resolution, and species specialisation affected SDM performance and reliability. We showed that species specialisation had by far the most dominant impact, where the algorithm performance and the effect of sample size and positional accuracy of species occurrences depended most on species specialisation (Fig 5). These conclusions are based on ecological reliability and spatial agreement, rather than statistical performance in modelling the data itself. The impact of grid resolution on the SDM’s reliability only became important with imprecise species occurrences when modelling highly specialized species (See S3 Appendix for more details on the impact of grid resolution on SDM). Our results also revealed that metrics of model performance can be misleading in representing the actual performance, if matching the true distribution was the goal.

We corroborate previous studies that found model performance and reliability improves with increasing sample size [15,16,35,38,39,78,95]. Nonetheless, useful distribution ranges for widespread and narrow-ranged species could be achieved with as few as 10 or 20 species occurrences respectively. In contrast to previous studies [15,16,35,38,39,41,96–103], our result indicates that the ranges of the less specialized species are in fact easier to predict than those of highly specialized species. These previous studies based their conclusions on SDM’s performance measured by the values of sensitivity and specificity, where the species ranges with low performance values were considered more difficult to predict. Together with other studies [80,104,105], we showed that this might not be always true, since SDM performance was strongly influenced by species specialisation and the size of the study area. Recently, a study evaluated SDM performance in response to the size of the buffer area (0–60%; 0% buffer, all background data were drawn from presence domain) surrounding the species range, and found that performance in ranges with buffer areas of ≤ 5% was no better than random, while increasing the size of the buffer area around the same range increased the performance value [48]. Accordingly, the evaluation metrics such as AUC and TSS could be more informing about how broadly the modelled species is distributed across the study area rather than inform about SDM performance [104,105]. Our results, in line with other studies [80,104,106,107], emphasize that standard evaluation metrics should not be used to compare performance between different species, nor within the same species when using different SDM settings

The minimum sample size required for a useful SDM varied according to species specialisation and the SDM algorithm. In generalist and restricted generalist species, both MAXENT and GLM predicted useful distribution ranges with as few as 10 species occurrences. In relaxed specialist and specialist species, the optimal minimum number was 20 using GBM and RF. While many studies have explored the impact of the number of species occurrences on SDM, until now this issue has remained unresolved. For example, using GARP, Stockwell and Peterson [35] suggested 10 species occurrences as minimum sample sizes, Papeş and Gaubert [37] suggested 15 using MAXENT, Drake *et al*. [36] suggested 40 using Support Vector Machine, and both Drake *et al*. [36] and Wisz *et al*. [38] suggested over 30 using GAM, GBM and MAXENT. Although many studies used the same algorithm, namely MAXENT, the results were inconsistent. This could be because the conclusions were based on SDM’s performance and ignored species specialisation. Only two studies considered species specialisation in their analyses, and both concluded that for narrow-ranged species, five species occurrences sufficed [15,16].

Several studies have shown that MAXENT stands out as the single best SDM algorithm [15,16,38,108], however, our results were not unanimous and revealed variation according to species specialisation and the number of occurrences. For example, at a high number of species occurrences (≥ 50) this algorithm outperformed the others across all species specialisation levels. At low number of occurrences (< 50), however, MAXENT underperformed in relaxed specialist and specialist species, and outperformed the other algorithms in generalist and restricted generalist species. We relate this variability to the differences in SDM algorithms, where, in contrast to the other algorithms, MAXENT and GLM had a tendency to over-predict (over-estimate the range occupied by a species) when fitted with a low number of occurrences (Figs E–L in S1 Appendix). As a result, the predicted ranges using MAXENT and GLM based on low numbers of occurrences resulted in a distribution range with a widespread probability surface (as a result of over-prediction). This widespread probability surface causes a good agreement with the “true” ranges for the wide spread species and poor agreement for the narrow-ranged species.

Our results revealed that the impact of the positional accuracy of species occurrences on SDM’s performance was relatively small across all species, where, in many cases, the models based on precise occurrences were only slightly better than those based on imprecise occurrences. These findings are in line with previous studies concluding that SDM is generally less sensitive to the levels of positional accuracy of species occurrences [12,43,44]. However, consulting the spatial agreement metrics revealed that the previous conclusion might not always be true, and that the sensitivity of SDM to the positional accuracy of species occurrences also depends on species specialisation and sample size. For generalist and restricted generalist species, the impact of species positional accuracy on SDM’s reliability was relatively small across all algorithms. However, the relaxed specialist and specialist species were in fact sensitive to the positional accuracy of the species occurrences. This sensitivity increased with increasing numbers of imprecise species occurrences and at low grid resolution. The sensitivity of specialised species to the level of positional accuracy could be due to the increased likelihood of assigning the imprecise species occurrences to unsuitable areas, whereas, in generalist species this likelihood is inherently lower. Accordingly, our results highlight the importance of investing time and effort into improving the positional accuracy of species occurrences for species with narrow distribution ranges, when modelling putative ecological specialists. However, for widespread species we believe that positional accuracy of species occurrence will have minimal effect on the reliability of SDM. This increases the relevance of data available in museums and online portals, especially for widespread species.

The sample size and positional accuracy of species occurrence data that can be used in SDM inherently varies according to the objective of the study. For example, if the goal is to define the environmental conditions that limit the distribution of a focal species, using high sample size and high positional accuracy may be necessary to minimize the commission error [109]. By contrast, if practitioners are interested in discovering a new population of a poorly known species, using high sample size and positional accuracy species occurrence data may not be crucially important. Therefore, defining the objective and the goal of the SDM is critical for achieving reliable conclusions in conjunction with a minimum amount of prior information about the species in question [109].

We have shown that species specialisation is the key factor with a dominant influence on SDM, which is usually unknown and/or not considered in species distribution models a-priori, while the spatial grid resolution has no considerable impact on SDM. We can conclude that narrow-ranged species are likely to be more sensitive than widespread species to changes in the level of positional accuracy of species occurrence and sample size. More important, we have also found that a high SDM performance does not always also imply a high reliability. In addition to our study and previous work, further effort needs to be directed towards investigating the impact of sample size and positional accuracy of species occurrences on: 1) SDM variables contribution, and 2) SDM transferability (spatial and temporal). Finally, it would be beneficial to explore the impact of using mixed levels of positional accuracy on SDM’s reliability. The work-flow (Fig 5) we provide should help other researchers to select the most appropriate approach according to the characteristic of the available data in the quest to make the best use of the data available in species distribution modelling studies.

## Supporting information

S1 Appendix Additional figures and tables. (DOC)

S2 Appendix Overall concordance correlation coefficient. (DOC)

S3 Appendix The impact of the grid resolutions on SDMs outcomes. (DOC)

## References

[1] Guillera-Arroita G, Lahoz-Monfort JJ, Elith J, Gordon A, Kujala H, Lentini PE, et al Is my species distribution model fit for purpose? Matching data and models to applications. Glob Ecol Biogeogr. 2015;24: 276–292. doi: 10.1111/geb.12268

[2] Guisan A, Thuiller W. Predicting species distribution: offering more than simple habitat models. Ecol Lett. 2005;8: 993–1009. doi: 10.1111/j.1461-0248.2005.00792.x 10.1111/j.1461-0248.2005.00792.x 34517687

[3] Guisan A, Zimmermann NE. Predictive habitat distribution models in ecology. Ecol Modell. 2000;135: 147–186. doi: 10.1016/S0304-3800(00)00354-9

[4] Elith J, Graham CH. Do they? How do they? WHY do they differ? On finding reasons for differing performances of species distribution models. Ecography (Cop). 2009;32: 66–77. doi: 10.1111/j.1600-0587.2008.05505.x

[5] Broennimann O, Guisan A. Predicting current and future biological invasions: both native and invaded ranges matter. Biol Lett. 2008;4: 585–589. doi: 10.1098/rsbl.2008.0254 18664415 10.1098/rsbl.2008.0254 PMC2610080

[6] Gallien L, Douzet R, Pratte S, Zimmermann NE, Thuiller W. Invasive species distribution models—how violating the equilibrium assumption can create new insights. Glob Ecol Biogeogr. 2012;21: 1126–1136. doi: 10.1111/j.1466-8238.2012.00768.x

[7] Thuiller W, Albert CH, Araújo MB, Berry PM, Cabeza M, Guisan A, et al Predicting global change impacts on plant species’ distributions: Future challenges. Perspect Plant Ecol Evol Syst. Elsevier; 2008;9: 137–152. doi: 10.1016/j.ppees.2007.09.004

[8] Li R, Tian H, Li X. Climate change induced range shifts of Galliformes in China. Integr Zool. 2010;5: 154–63. doi: 10.1111/j.1749-4877.2010.00198.x 21392333 10.1111/j.1749-4877.2010.00198.x

[9] Marini MÂ, Barbet-Massin M, Lopes LE, Jiguet F. Major current and future gaps of Brazilian reserves to protect Neotropical savanna birds. Biol Conserv. 2009;142: 3039–3050. doi: 10.1016/j.biocon.2009.08.002

[10] Schadt S, Revilla E, Wiegand T, Knauer F, Kaczensky P, Breitenmoser U, et al Assessing the suitability of central European landscapes for the reintroduction of Eurasian lynx. J Appl Ecol. 2002;39: 189–203. doi: 10.1046/j.1365-2664.2002.00700.x

[11] Marini MÂ, Barbet-Massin M, Martinez J, Prestes NP, Jiguet F. Applying ecological niche modelling to plan conservation actions for the Red-spectacled Amazon (*Amazona pretrei*). Biol Conserv. 2010;143: 102–112. doi: 10.1016/j.biocon.2009.09.009

[12] Graham CH, Elith J, Hijmans RJ, Guisan A, Townsend Peterson A, Loiselle BA. The influence of spatial errors in species occurrence data used in distribution models. J Appl Ecol. 2008;45: 239–247. doi: 10.1111/j.1365-2664.2007.01408.x

[13] Wieczorek J, Guo Q, Hijmans R. The point-radius method for georeferencing locality descriptions and calculating associated uncertainty. Int J Geogr Inf Sci. Taylor & Francis Group; 2004;18: 745–767. doi: 10.1080/13658810412331280211

[14] Tessarolo G, Rangel TF, Araújo MB, Hortal J. Uncertainty associated with survey design in Species Distribution Models. Divers Distrib. 2014;20: 1258–1269. doi: 10.1111/ddi.12236

[15] Hernandez PA, Graham CH, Master LL, Albert DL. The effect of sample size and species characteristics on performance of different species distribution modeling methods. Ecography (Cop). 2006;29: 773–785. doi: 10.1111/j.0906-7590.2006.04700.x

[16] van Proosdij ASJ, Sosef MSM, Wieringa JJ, Raes N. Minimum required number of specimen records to develop accurate species distribution models. Ecography (Cop). 2016;39: 542–552. doi: 10.1111/ecog.01509

[17] Naimi B, Skidmore AK, Groen TA, Hamm NAS. Spatial autocorrelation in predictors reduces the impact of positional uncertainty in occurrence data on species distribution modelling. J Biogeogr. 2011;38: 1497–1509. doi: 10.1111/j.1365-2699.2011.02523.x

[18] Naimi B, Hamm NAS, Groen TA, Skidmore AK, Toxopeus AG. Where is positional uncertainty a problem for species distribution modelling? Ecography (Cop). 2014;37: 191–203. doi: 10.1111/j.1600-0587.2013.00205.x

[19] Rocchini D, Hortal J, Lengyel S, Lobo JM, Jimenez-Valverde A, Ricotta C, et al Accounting for uncertainty when mapping species distributions: The need for maps of ignorance. Prog Phys Geogr. SAGE Publications; 2011;35: 211–226. doi: 10.1177/0309133311399491

[20] Costa H, Foody G, Jiménez S, Silva L. Impacts of Species Misidentification on Species Distribution Modeling with Presence-Only Data. ISPRS Int J Geo-Information. Multidisciplinary Digital Publishing Institute; 2015;4: 2496–2518. doi: 10.3390/ijgi4042496

[21] Miller DA, Nichols JD, McClintock BT, Grant EHC, Bailey LL, Weir LA. Improving occupancy estimation when two types of observational error occur: non-detection and species misidentification. Ecology. Ecological Society of America; 2011;92: 1422–1428. doi: 10.1890/10-1396.1 21870616 10.1890/10-1396.1

[22] Scott WA, Hallam CJ. Assessing species misidentification rates through quality assurance of vegetation monitoring. Plant Ecol. Kluwer Academic Publishers; 2003;165: 101–115. doi: 10.1023/A:1021441331839

[23] Syfert MM, Smith MJ, Coomes DA. The effects of sampling bias and model complexity on the predictive performance of MaxEnt species distribution models. PLoS One. Public Library of Science; 2013;8: e55158 doi: 10.1371/journal.pone.0055158 23457462 10.1371/journal.pone.0055158 PMC3573023

[24] Fourcade Y, Engler JO, Rödder D, Secondi J. Mapping species distributions with MAXENT using a geographically biased sample of presence data: a performance assessment of methods for correcting sampling bias. PLoS One. Public Library of Science; 2014;9: e97122 doi: 10.1371/journal.pone.0097122 24818607 10.1371/journal.pone.0097122 PMC4018261

[25] Kramer-Schadt S, Niedballa J, Pilgrim JD, Schröder B, Lindenborn J, Reinfelder V, et al The importance of correcting for sampling bias in MaxEnt species distribution models. Divers Distrib. 2013;19: 1366–1379. doi: 10.1111/ddi.12096

[26] Phillips SJ, Dudík M, Elith J, Graham CH, Lehmann A, Leathwick J, et al Sample selection bias and presence-only distribution models: implications for background and pseudo-absence data. Ecol Appl. Ecological Society of America; 2009;19: 181–197. doi: 10.1890/07-2153.1 19323182 10.1890/07-2153.1

[27] Barbet-Massin M, Jiguet F, Albert CH, Thuiller W. Selecting pseudo-absences for species distribution models: how, where and how many? Methods Ecol Evol. 2012;3: 327–338. doi: 10.1111/j.2041-210X.2011.00172.x

[28] Dudík M, Phillips SJ, Schapire RE. Correcting sample selection bias in maximum entropy density estimation Advances in Neural Information Processing Systems. The MIT Press; 2005 pp. 323–330. Available: http://machinelearning.wustl.edu/mlpapers/papers/NIPS2005_243

[29] Fithian W, Elith J, Hastie T, Keith DA. Bias correction in species distribution models: pooling survey and collection data for multiple species. Methods Ecol Evol. 2015;6: 424–438. doi: 10.1111/2041-210X.12242 27840673 10.1111/2041-210X.12242 PMC5102514

[30] Hortal J, Jiménez-Valverde A, Gómez JF, Lobo JM, Baselga A. Historical bias in biodiversity inventories affects the observed environmental niche of the species. Oikos. 2008;117: 847–858. doi: 10.1111/j.0030-1299.2008.16434.x

[31] Loiselle BA, Jørgensen PM, Consiglio T, Jiménez I, Blake JG, Lohmann LG, et al Predicting species distributions from herbarium collections: does climate bias in collection sampling influence model outcomes? J Biogeogr. 2008;35: 105–116. doi: 10.1111/j.1365-2699.2007.01779.x

[32] Monk J. How long should we ignore imperfect detection of species in the marine environment when modelling their distribution? Fish Fish. 2014;15: 352–358. doi: 10.1111/faf.12039

[33] Osborne PE, Suárez-Seoane S. Should data be partitioned spatially before building large-scale distribution models? Ecol Modell. 2002;157: 249–259. doi: 10.1016/S0304-3800(02)00198-9

[34] Phillips SJ. Transferability, sample selection bias and background data in presence-only modelling: a response to Peterson et al. (2007). Ecography (Cop). 2008;31: 272–278. doi: 10.1111/j.0906-7590.2008.5378.x

[35] Stockwell DR., Peterson AT. Effects of sample size on accuracy of species distribution models. Ecol Modell. 2002;148: 1–13. doi: 10.1016/S0304-3800(01)00388-X

[36] Drake JM, Randin C, Guisan A. Modelling ecological niches with support vector machines. J Appl Ecol. Blackwell Publishing Ltd; 2006;43: 424–432. doi: 10.1111/j.1365-2664.2006.01141.x

[37] Papeş M, Gaubert P. Modelling ecological niches from low numbers of occurrences: assessment of the conservation status of poorly known viverrids (Mammalia, Carnivora) across two continents. Divers Distrib. 2007;13: 890–902. doi: 10.1111/j.1472-4642.2007.00392.x

[38] Wisz MS, Hijmans RJ, Li J, Peterson AT, Graham CH, Guisan A. Effects of sample size on the performance of species distribution models. Divers Distrib. 2008;14: 763–773. doi: 10.1111/j.1472-4642.2008.00482.x

[39] Mateo RG, Felicísimo ÁM, Muñoz J. Effects of the number of presences on reliability and stability of MARS species distribution models: the importance of regional niche variation and ecological heterogeneity. J Veg Sci. 2010;21: 908–922. doi: 10.1111/j.1654-1103.2010.01198.x

[40] Marini MÂ, Barbet-Massin M, Lopes LE, Jiguet F. Predicting the occurrence of rare Brazilian birds with species distribution models. J Ornithol. 2010;151: 857–866. doi: 10.1007/s10336-010-0523-y

[41] McPherson JM, Jetz W, Rogers DJ. The effects of species’ range sizes on the accuracy of distribution models: ecological phenomenon or statistical artefact? J Appl Ecol. 2004;41: 811–823. doi: 10.1111/j.0021-8901.2004.00943.x

[42] Fernández M, Blum S, Reichle S, Guo Q, Holzman B, Hamilton H. Locality uncertainty and the differential performance of four common niche-based. modeling techniques. Biodivers Informatics. 2009;6: 36–52. doi: 10.17161/bi.v6i1.3314

[43] Osborne PE, Leitão PJ. Effects of species and habitat positional errors on the performance and interpretation of species distribution models. Divers Distrib. 2009;15: 671–681. doi: 10.1111/j.1472-4642.2009.00572.x

[44] Hayes MA, Ozenberger K, Cryan PM, Wunder MB. Not to Put Too Fine a Point on It—Does Increasing Precision of Geographic Referencing Improve Species Distribution Models for a Wide-Ranging Migratory Bat? Acta Chiropterologica. Museum and Institute of Zoology at the Polish Academy of Science; 2015;17: 159–169. doi: 10.3161/15081109ACC2015.17.1.013

[45] Mitchell PJ, Monk J, Laurenson L. Sensitivity of fine-scale species distribution models to locational uncertainty in occurrence data across multiple sample sizes. Chisholm R, editor. Methods Ecol Evol. 2017;8: 12–21. doi: 10.1111/2041-210X.12645

[46] Visscher D.R. GPS measurement error and resource selection functions in a fragmented landscape. Ecography (Cop). 2006;29: 458–464. doi: 10.1111/j.0906-7590.2006.04648.x

[47] Johnson CJ, Gillingham MP. Sensitivity of species-distribution models to error, bias, and model design: An application to resource selection functions for woodland caribou. Ecol Modell. 2008;213: 143–155. doi: 10.1016/j.ecolmodel.2007.11.013

[48] Barve N, Barve V, Jiménez-Valverde A, Lira-Noriega A, Maher SP, Peterson AT, et al The crucial role of the accessible area in ecological niche modeling and species distribution modeling. Ecol Modell. 2011;222: 1810–1819. doi: 10.1016/j.ecolmodel.2011.02.011

[49] Boucher-Lalonde V, Morin A, Currie DJ. A consistent occupancy-climate relationship across birds and mammals of the Americas. Oikos. 2014; no-no. doi: 10.1111/oik.01277

[50] Boucher-Lalonde V, Morin A, Currie DJ. How are tree species distributed in climatic space? A simple and general pattern. Glob Ecol Biogeogr. 2012;21: 1157–1166. doi: 10.1111/j.1466-8238.2012.00764.x

[51] Meynard CN, Quinn JF. Predicting species distributions: a critical comparison of the most common statistical models using artificial species. J Biogeogr. 2007;34: 1455–1469. doi: 10.1111/j.1365-2699.2007.01720.x

[52] Leroy B, Meynard CN, Bellard C, Courchamp F. virtualspecies, an R package to generate virtual species distributions. Ecography (Cop). 2016;39: 599–607. doi: 10.1111/ecog.01388

[53] Jiménez-Valverde A, Lobo JM, Hortal J. The effect of prevalence and its interaction with sample size on the reliability of species distribution models. Community Ecol. 2009;10: 196–205. Available: http://www.jstor.org/stable/24113558

[54] Varela S, Anderson RP, García-Valdés R, Fernández-González F. Environmental filters reduce the effects of sampling bias and improve predictions of ecological niche models. Ecography (Cop). Blackwell Publishing Ltd; 2014;37: 1084–1091. doi: 10.1111/j.1600-0587.2013.00441.x

[55] Hirzel A, Guisan A. Which is the optimal sampling strategy for habitat suitability modelling. Ecol Modell. 2002;157: 331–341. doi: 10.1016/S0304-3800(02)00203-X

[56] Broennimann O, Fitzpatrick MC, Pearman PB, Petitpierre B, Pellissier L, Yoccoz NG, et al Measuring ecological niche overlap from occurrence and spatial environmental data. Glob Ecol Biogeogr. Blackwell Publishing Ltd; 2012;21: 481–497. doi: 10.1111/j.1466-8238.2011.00698.x

[57] Miller JA. Virtual species distribution models: Using simulated data to evaluate aspects of model performance. Prog Phys Geogr. 2014;38: 117–128. doi: 10.1177/0309133314521448

[58] Prasad AM, Iverson LR, Liaw A. Newer Classification and Regression Tree Techniques: Bagging and Random Forests for Ecological Prediction. Ecosystems. Springer-Verlag; 2006;9: 181–199. doi: 10.1007/s10021-005-0054-1

[59] Meynard CN, Kaplan DM. Using virtual species to study species distributions and model performance. Silman M, editor. J Biogeogr. 2013;40: 1–8. doi: 10.1111/jbi.12006

[60] Hirzel AH, Helfer V, Metral F. Assessing habitat-suitability models with a virtual species. Ecol Modell. 2001;145: 111–121. doi: 10.1016/S0304-3800(01)00396-9

[61] García Molinos J, Halpern BS, Schoeman DS, Brown CJ, Kiessling W, Moore PJ, et al Climate velocity and the future global redistribution of marine biodiversity. Nat Clim Chang. Nature Research; 2015;6: 83–88. doi: 10.1038/nclimate2769

[62] Li W, Guo Q, Elkan C. Can we model the probability of presence of species without absence data? Ecography (Cop). 2011;34: 1096–1105. doi: 10.1111/j.1600-0587.2011.06888.x

[63] Santika T. Assessing the effect of prevalence on the predictive performance of species distribution models using simulated data. Glob Ecol Biogeogr. 2011;20: 181–192. doi: 10.1111/j.1466-8238.2010.00581.x

[64] Hefley TJ, Baasch DM, Tyre AJ, Blankenship EE. Correction of location errors for presence-only species distribution models. Warton D, editor. Methods Ecol Evol. 2014;5: 207–214. doi: 10.1111/2041-210X.12144

[65] Quinn GP, Keough MJ. Experimental design and data analysis for biologists. NY, U.S.A.: Cambridge University Press; 2002.

[66] Naimi B. usdm: Uncertainty Analysis for Species Distribution Models. R package version 1.1–15. [Internet]. 2015. Available: https://cran.r-project.org/package=usdm

[67] Duque-Lazo J, van Gils H, Groen TA, Navarro-Cerrillo RM. Transferability of species distribution models: The case of *Phytophthora cinnamomi* in Southwest Spain and Southwest Australia. Ecol Modell. 2016;320: 62–70. doi: 10.1016/j.ecolmodel.2015.09.019

[68] Phillips SJ, Anderson RP, Schapire RE. Maximum entropy modeling of species geographic distributions. Ecol Modell. 2006;190: 231–259. doi: 10.1016/j.ecolmodel.2005.03.026

[69] McCullagh, Peter; Nelder J. Generalized Linear Models. Second Boca Raton: Chapman and Hall; 1989.

[70] Austin M. Spatial prediction of species distribution: an interface between ecological theory and statistical modelling. Ecol Modell. 2002;157: 101–118. doi: 10.1016/S0304-3800(02)00205-3

[71] Hastie T. and Tibshirani R. Generalized Additive Models. 1st ed. London: Chapman & Hall/CRC; 1990.

[72] Elith J, Leathwick JR, Hastie T. A working guide to boosted regression trees. J Anim Ecol. 2008;77: 802–813. doi: 10.1111/j.1365-2656.2008.01390.x 18397250 10.1111/j.1365-2656.2008.01390.x

[73] Breiman L. Statistical Modeling: The Two Cultures (with comments and a rejoinder by the author). Stat Sci. Institute of Mathematical Statistics; 2001;16: 199–231. Available: http://projecteuclid.org/euclid.ss/1009213726

[74] Phillips SJ, Dudík M. Modeling of species distributions with Maxent: new extensions and a comprehensive evaluation. Ecography (Cop). 2008;31: 161–175. doi: 10.1111/j.0906-7590.2008.5203.x

[75] Thuiller W, Georges D, Engler R. biomod2: Ensemble platform for species distribution modeling.R package version 3.3-13/r726. [Internet]. 2016. Available: https://r-forge.r-project.org/projects/biomod/

[76] Anderson RP, Gonzalez I. Species-specific tuning increases robustness to sampling bias in models of species distributions: An implementation with Maxent. Ecol Modell. 2011;222: 2796–2811. doi: 10.1016/j.ecolmodel.2011.04.011

[77] Baldwin RA. Use of Maximum Entropy Modeling in Wildlife Research. Entropy. Molecular Diversity Preservation International; 2009;11: 854–866. doi: 10.3390/e11040854

[78] Pearce J, Ferrier S. Evaluating the predictive performance of habitat models developed using logistic regression. Ecol Modell. 2000;133: 225–245. doi: 10.1016/S0304-3800(00)00322-7

[79] Liu C, White M, Newell G. Measuring and comparing the accuracy of species distribution models with presence-absence data. Ecography (Cop). 2011;34: 232–243. doi: 10.1111/j.1600-0587.2010.06354.x

[80] Jiménez-Valverde A. Insights into the area under the receiver operating characteristic curve (AUC) as a discrimination measure in species distribution modelling. Glob Ecol Biogeogr. 2012;21: 498–507. doi: 10.1111/j.1466-8238.2011.00683.x

[81] Allouche O, Tsoar A, Kadmon R. Assessing the accuracy of species distribution models: prevalence, kappa and the true skill statistic (TSS). J Appl Ecol. 2006;43: 1223–1232. doi: 10.1111/j.1365-2664.2006.01214.x

[82] Manel S, Williams HC, Ormerod SJ. Evaluating presence-absence models in ecology: the need to account for prevalence. J Appl Ecol. 2002;38: 921–931. doi: 10.1046/j.1365-2664.2001.00647.x

[83] Franklin J. Mapping species distributions: spatial inference and prediction. Cambridge: Cambridge University Press; 2010.

[84] González-Irusta JM, González-Porto M, Sarralde R, Arrese B, Almón B, Martín-Sosa P. Comparing species distribution models: a case study of four deep sea urchin species. Hydrobiologia. 2014;745: 43–57. doi: 10.1007/s10750-014-2090-3

[85] Lahoz-Monfort JJ, Guillera-Arroita G, Wintle BA. Imperfect detection impacts the performance of species distribution models. Glob Ecol Biogeogr. 2014;23: 504–515. doi: 10.1111/geb.12138

[86] VanDerWal J, Falconi L, Januchowski S, Shoo L, Storlie C. SDMTools: Species Distribution Modelling Tools: Tools for processing data associated with species distribution modelling exercises. R package version 1.1–221. [Internet]. 2014. Available: http://www.rforge.net/SDMTools/

[87] R Core Team. R: A language and environment for statistical computing. Vienna, Austria: R Foundation for Statistical Computing. Available at: http://www.R-project.org/ [Internet]. Vienna, Austria: R Foundation for Statistical Computing; 2016. Available: http://www.r-project.org/

[88] Akaike H. Information theory as an extension of the maximum likelihood principle In: Parzen E, Tanabe K, Kitagawa G, editors. Selected Papers of Hirotugu Akaike. Springer New York; 1998 pp. 199–213.

[89] Schoener TW. The Anolis Lizards of Bimini: Resource Partitioning in a Complex Fauna. Ecol Soc Am. 1968;49: 704–726. doi: 10.2307/1935534

[90] Hijmans RJ, Phillips S, John Leathwick, Elith J. Dismo: Species distribution modeling. R package version 1.1–1. [Internet]. 2016. Available: https://cran.r-project.org/package=dismo

[91] Warren DL, Glor RE, Turelli M. Environmental niche equivalency versus conservatism: quantitative approaches to niche evolution. Evolution. 2008;62: 2868–83. doi: 10.1111/j.1558-5646.2008.00482.x 18752605 10.1111/j.1558-5646.2008.00482.x

[92] Faleiro FV, Silva DP, de Carvalho RA, Särkinen T, De Marco P. Ring out the bells, we are being invaded! Niche conservatism in exotic populations of the Yellow Bells, Tecoma stans (Bignoniaceae). Nat Conserv. 2015;13: 24–29. doi: 10.1016/j.ncon.2015.04.004

[93] Barnhart HX, Haber M, Song J. Overall Concordance Correlation Coefficient for Evaluating Agreement Among Multiple Observers. Biometrics. 2002;58: 1020–1027. doi: 10.1111/j.0006-341X.2002.01020.x 12495158 10.1111/j.0006-341x.2002.01020.x

[94] Stevenson M, Nunes T, Heuer C, Marshall J, Sanchez J, Thornton R, et al. epiR: Tools for the Analysis of Epidemiological Data. R package version 0.9–69. 2015.

[95] Cumming GS. Using between-model comparisons to fine-tune linear models of species ranges. J Biogeogr. 2000;27: 441–455. doi: 10.1046/j.1365-2699.2000.00408.x

[96] Gonzalez SC, Soto-Centeno JA, Reed DL, Peterson A, Sánchez-Cordero V, Beard C, et al Population distribution models: species distributions are better modeled using biologically relevant data partitions. BMC Ecol. BioMed Central; 2011;11: 20 doi: 10.1186/1472-6785-11-20 21929792 10.1186/1472-6785-11-20 PMC3184255

[97] Grenouillet G, Buisson L, Casajus N, Lek S. Ensemble modelling of species distribution: the effects of geographical and environmental ranges. Ecography (Cop). Blackwell Publishing Ltd; 2011;34: 9–17. doi: 10.1111/j.1600-0587.2010.06152.x

[98] Brotons L, Thuiller W, Araújo MB, Hirzel AH. Presence-absence versus presence-only modelling methods for predicting bird habitat suitability. Ecography (Cop). 2004;27: 437–448. doi: 10.1111/j.0906-7590.2004.03764.x

[99] Segurado P, Araújo MB. An evaluation of methods for modelling species distributions. J Biogeogr. 2004;31: 1555–1568. doi: 10.1111/j.1365-2699.2004.01076.x

[100] McPherson J M., Jetz W. Effects of species’ ecology on the accuracy of distribution models. Ecography (Cop). 2007;30: 135–151. doi: 10.1111/j.0906-7590.2007.04823.x

[101] Luoto M, Pöyry J, Heikkinen RK, Saarinen K. Uncertainty of bioclimate envelope models based on the geographical distribution of species. Glob Ecol Biogeogr. Blackwell Science Ltd; 2005;14: 575–584. doi: 10.1111/j.1466-822X.2005.00186.x

[102] Araújo MB, Williams PH. Selecting areas for species persistence using occurrence data. Biol Conserv. 2000;96: 331–345. doi: 10.1016/S0006-3207(00)00074-4

[103] Karl JW, Heglund PJ, Garton EO, Scott JM, Wright NM, Hutto RL. Sensitivity of Species Habitat-Relationship Model Performance to Factors of Scale. Ecol Appl. Ecological Society of America; 2000;10: 1690–1705. doi: 10.1890/1051-0761(2000)010[1690:SOSHRM]2.0.CO;2

[104] Lobo JM, Jiménez-Valverde A, Real R. AUC: a misleading measure of the performance of predictive distribution models. Glob Ecol Biogeogr. 2008;17: 145–151. doi: 10.1111/j.1466-8238.2007.00358.x

[105] Ruete A, Leynaud GC. Goal-oriented evaluation of species distribution models’ accuracy and precision: True Skill Statistic profile and uncertainty maps. PeerJ Prepr. PeerJ Inc.; 2015;3: e1478 doi: 10.7287/peerj.preprints.1208v1

[106] Merow C, Smith MJ, Silander JA. A practical guide to MaxEnt for modeling species’ distributions: what it does, and why inputs and settings matter. Ecography (Cop). 2013;36: 1058–1069. doi: 10.1111/j.1600-0587.2013.07872.x

[107] Peterson AT, Papeş M, Soberón J. Rethinking receiver operating characteristic analysis applications in ecological niche modeling. Ecol Modell. 2008;213: 63–72. doi: 10.1016/j.ecolmodel.2007.11.008

[108] Elith J, Graham CH, P. Anderson R, Dudík M, Ferrier S, Guisan A, et al Novel methods improve prediction of species’ distributions from occurrence data. Ecography (Cop). 2006;29: 129–151. doi: 10.1111/j.2006.0906–7590.04596.x

[109] Araújo MB, Peterson AT. Uses and misuses of bioclimatic envelope modeling. Ecology. 2012;93: 1527–1539. doi: 10.1890/11-1930.1 22919900 10.1890/11-1930.1

