# Supplementary material for: The interplay of various sources of noise on reliability of species distribution models hinges on ecological specialisation
Source: PLoS One. 2017 Nov 13;12(11):e0187906. doi: 10.1371/journal.pone.0187906 (PMC5683637; doi:10.1371/journal.pone.0187906)
Supplement: S1 Appendix — (DOC) [file pone.0187906.s001.doc]

**S1 Appendix: Additional figures and tables**


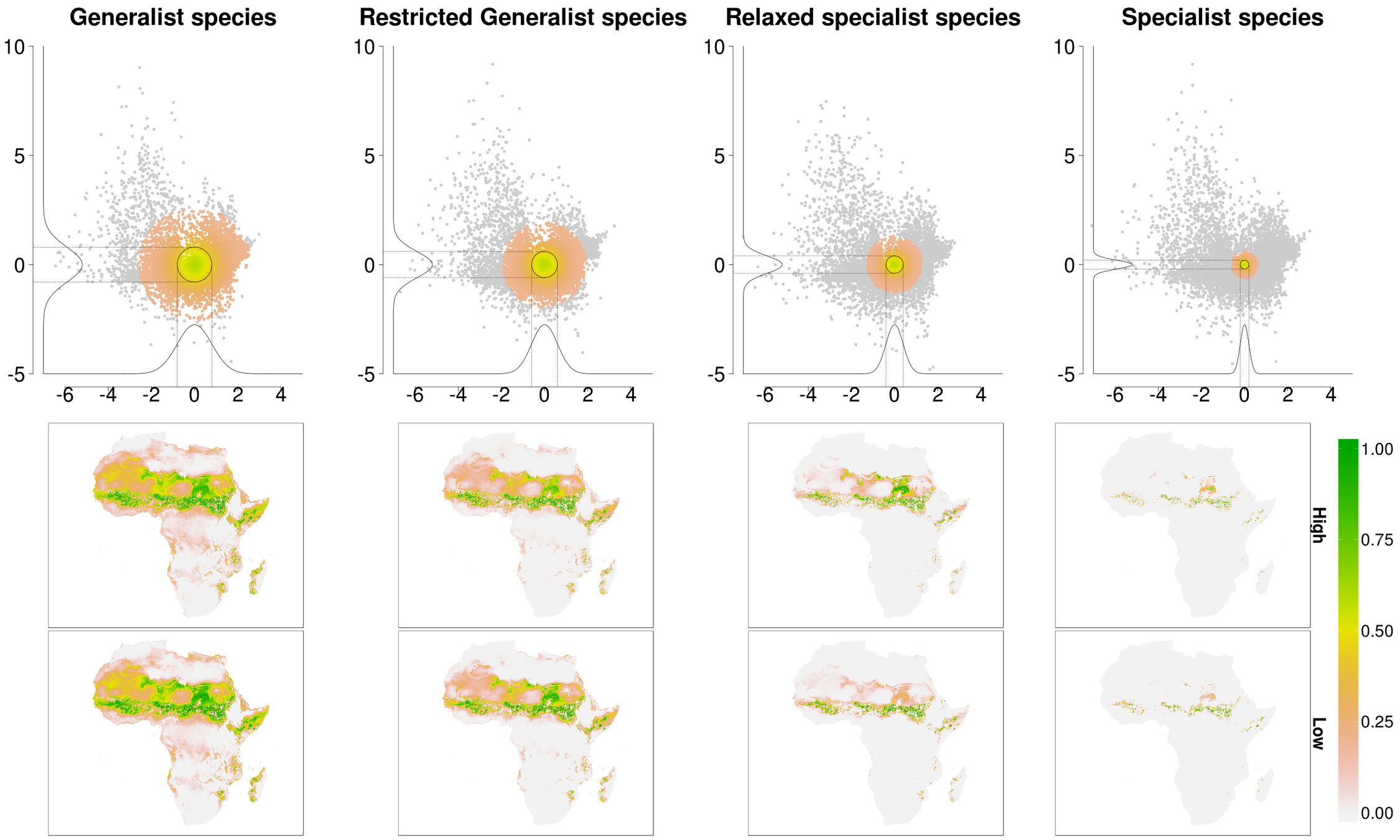
**Figure A: The continuous suitability maps “true distribution ranges” for four species at high and low grid resolutions.** The first row shows the environmental suitability determined by applying Gaussian distribution function one each of the first two axes of the PCA, the first PCA’s axis is plotted on x axis and the second PCA’s axis is plotted on the y axis. The width of the bell curve is determined by adjusting the standard deviation corresponding to the ecological specialisation of the species. The second and third rows show the geographic suitability for the four species over Africa using high spatial resolution (High 2.5 arc-min) and low spatial resolution (Low 10 arc-min) respectively. The coloured scale bar on the right side shows suitability values from 0 to 1, where 0 represents highly unsuitable conditions and 1 highly suitable conditions.


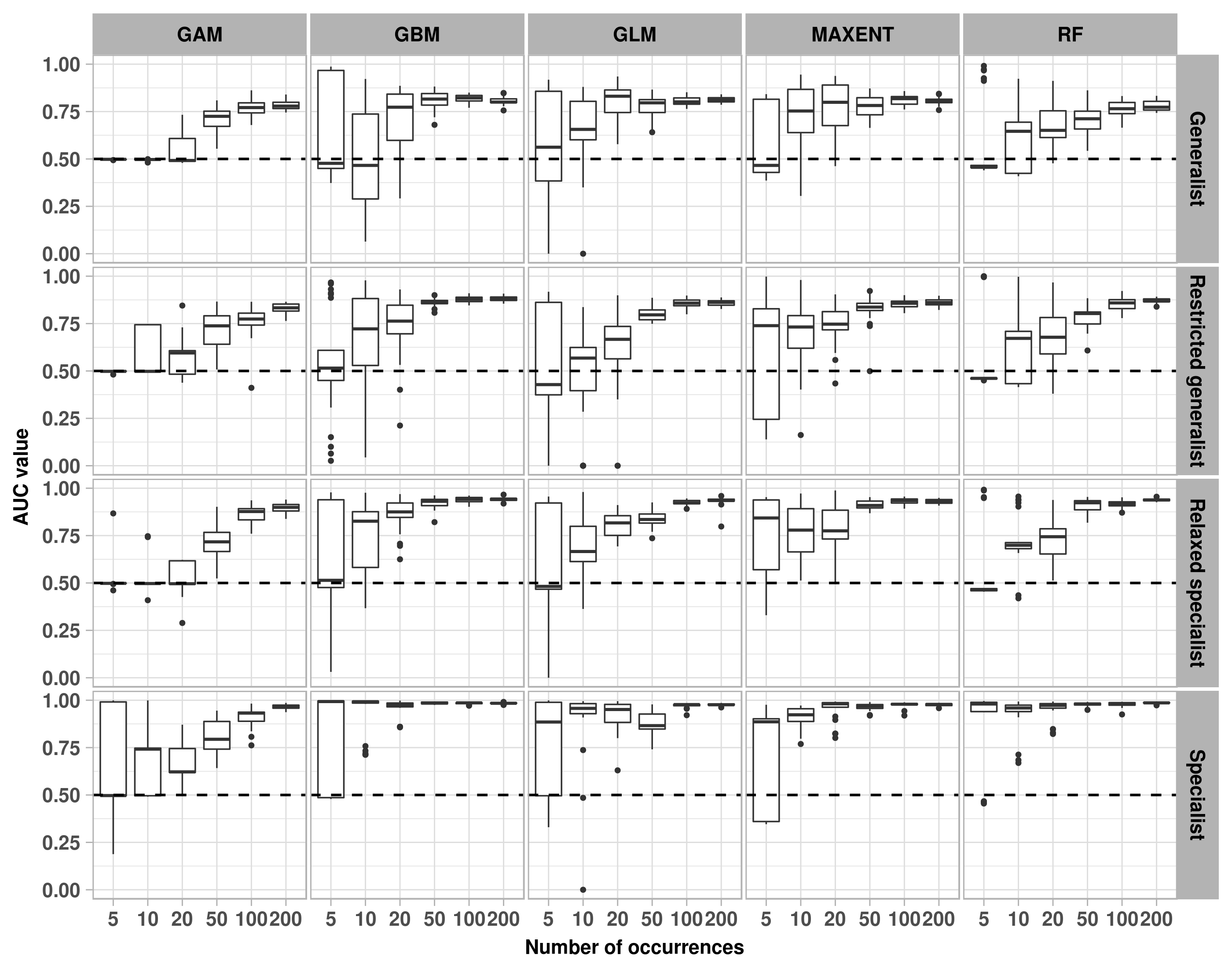
**Figure B: The inter-quantile range of the standard AUC values across species and algorithms at low grid resolution.** This plot shows the variation in model performance for four species (row-wise) with increasing the sample size (x axis) using five different SDM algorithms (column-wise). The dashed line represents the threshold line, where median values above this line indicate good performance.


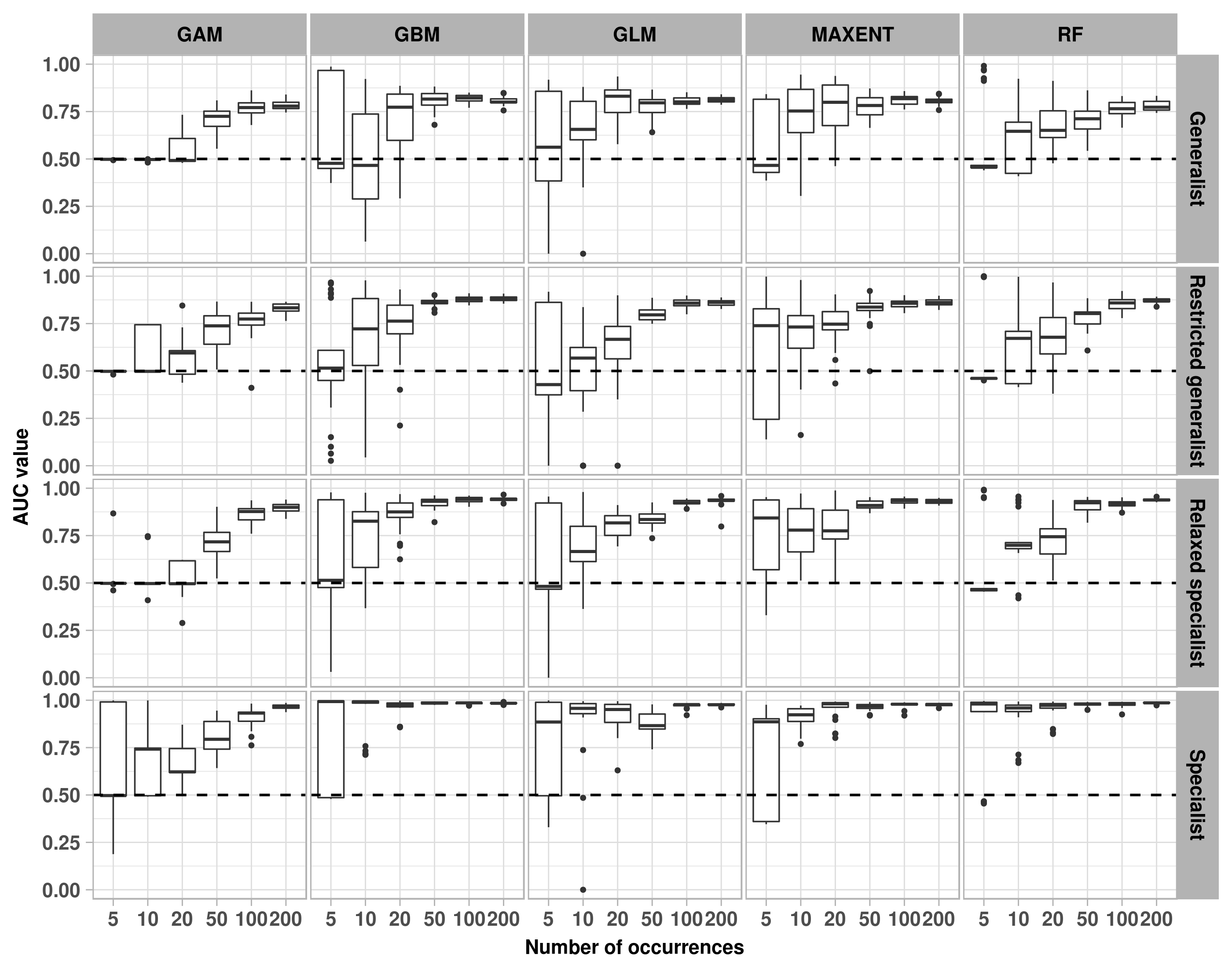
**Figure C: The inter-quantile range of the standard AUC values across species and algorithms at high grid resolution**. This plot shows the variation in model performance for four species (row-wise) with increasing the sample size (x axis) using five different SDM algorithms (column-wise). The dashed line represents the threshold line, where median values above this line indicate good performance.


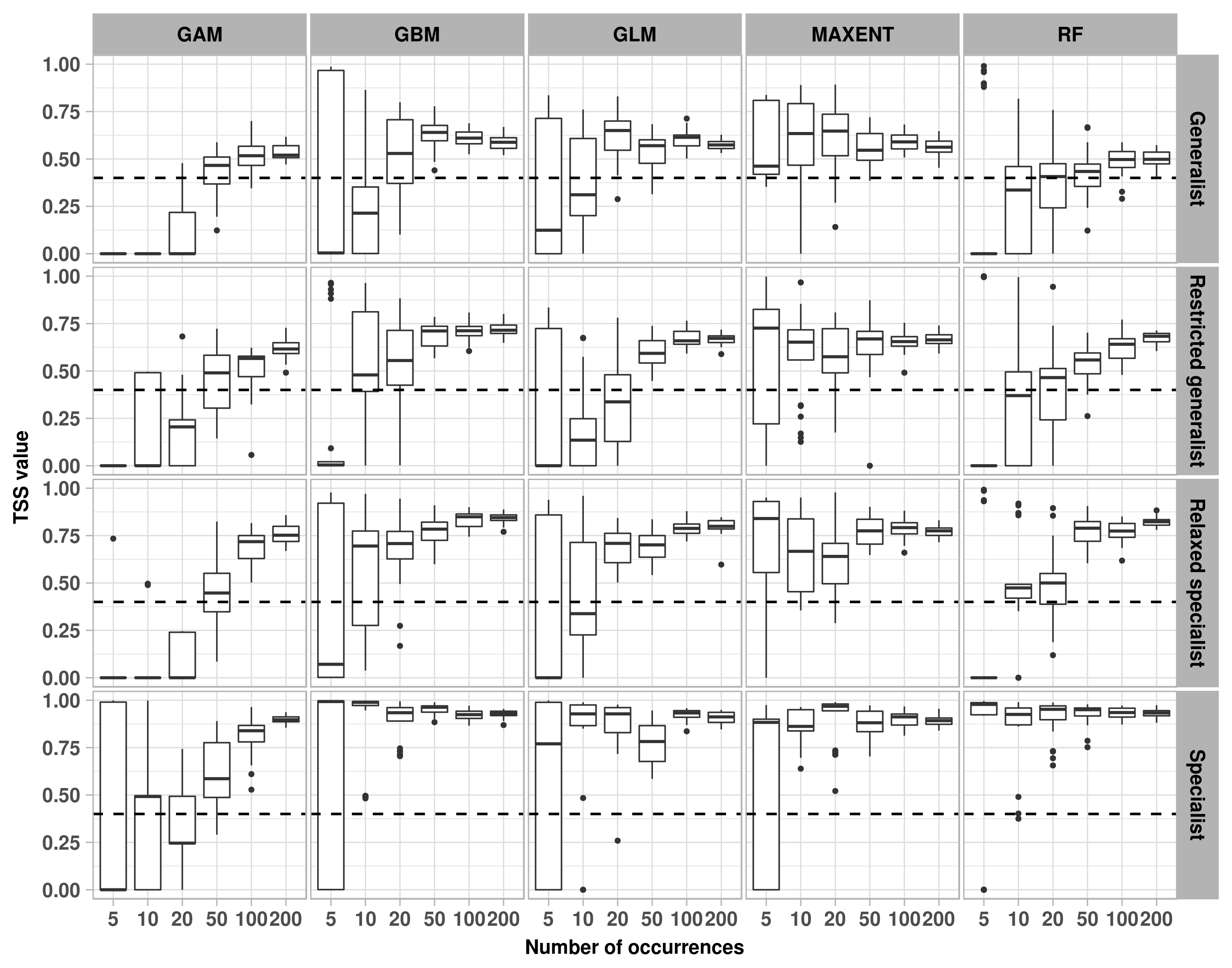
**Figure D: The inter-quantile range of the standard TSS values across species and algorithms at low grid resolution.** This plot shows the variation in model performance for four species (row-wise) with increasing the sample size (x axis) using five different SDM algorithms (column-wise). The dashed line represents the threshold line, where median values above this line indicate good performance.


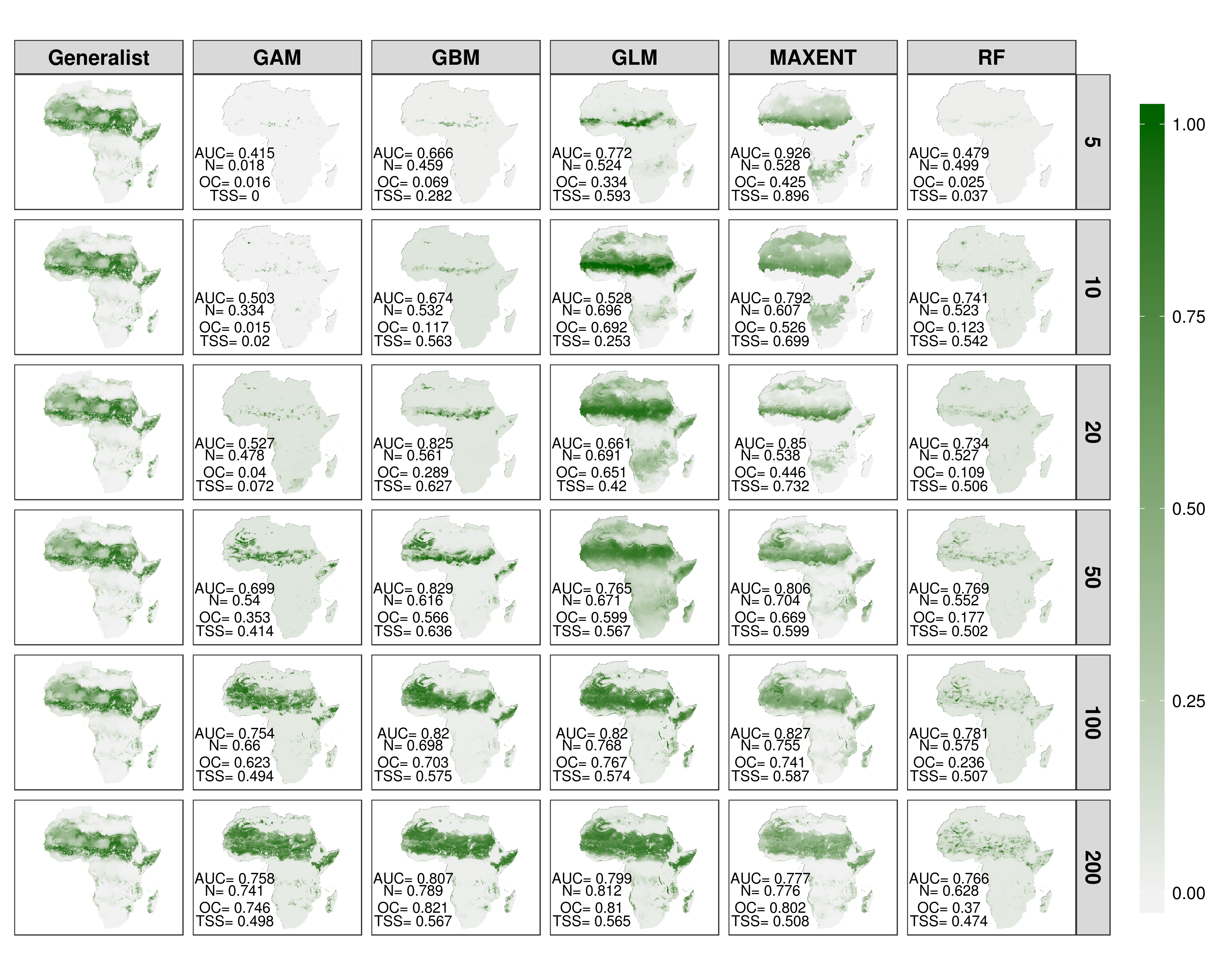
**Figure E: Spatial agreement between the predicted and “true” ranges for the generalist species (low grid resolution).** The Y axis represents the number of species occurrences and the X axis is the model algorithms. Each plot has the value of AUC and TSS and the N stand for Niche overlap and OC stands for OCCC. The left column is the “true” ranges and the right columns are the predicted ones. The scale bar in the right side represents the suitability from 0 to 1.


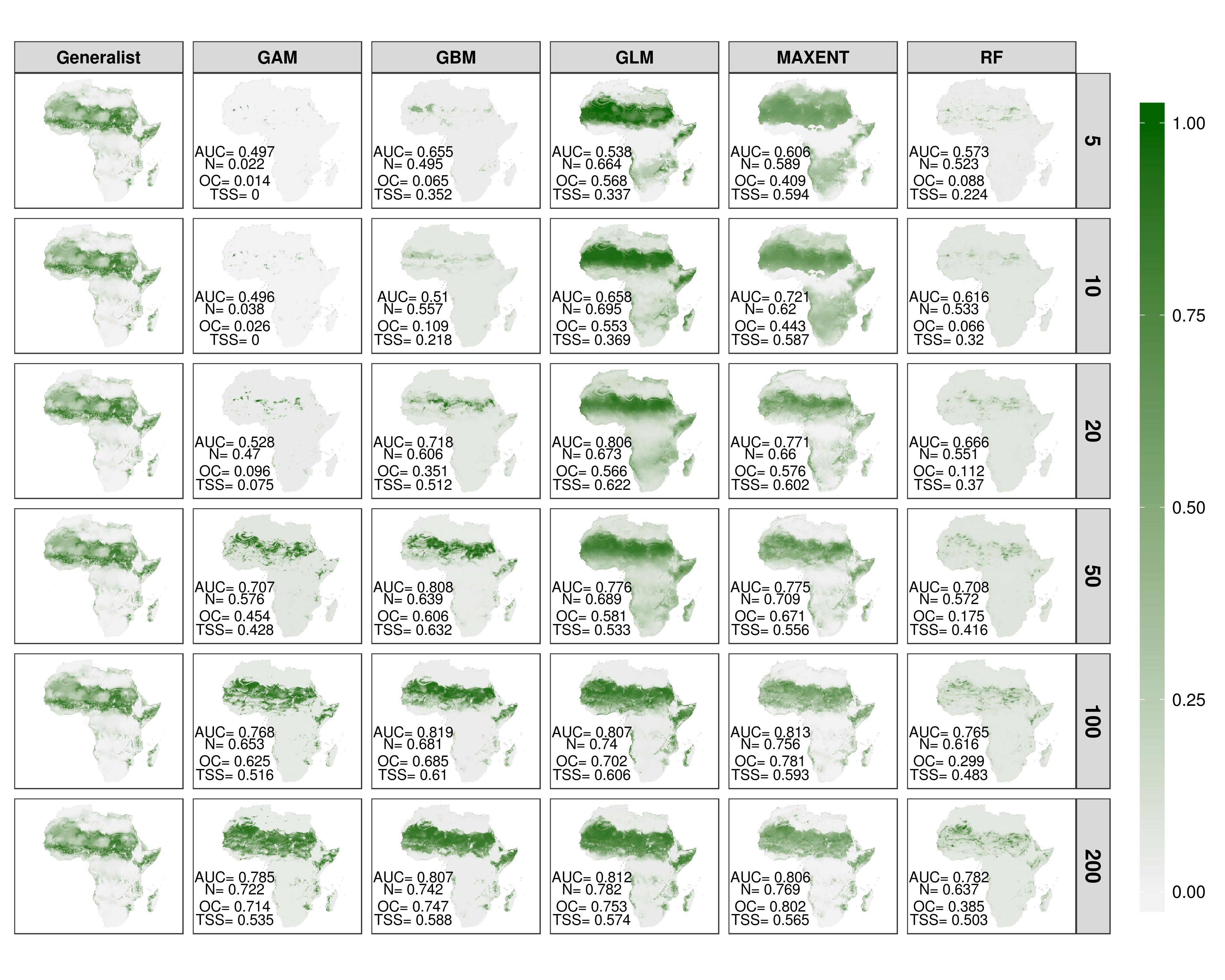
**Figure F: Spatial agreement between the predicted and “true” ranges for the generalist species (high grid resolution).** The Y axis represents the number of species occurrences and the X axis is the model algorithms. Each plot has the value of AUC and TSS and the N stand for Niche overlap and OC stands for OCCC. The left column is the “true” ranges and the right columns are the predicted ones. The colour scale bar in the right side represents the suitability from 0 to 1.


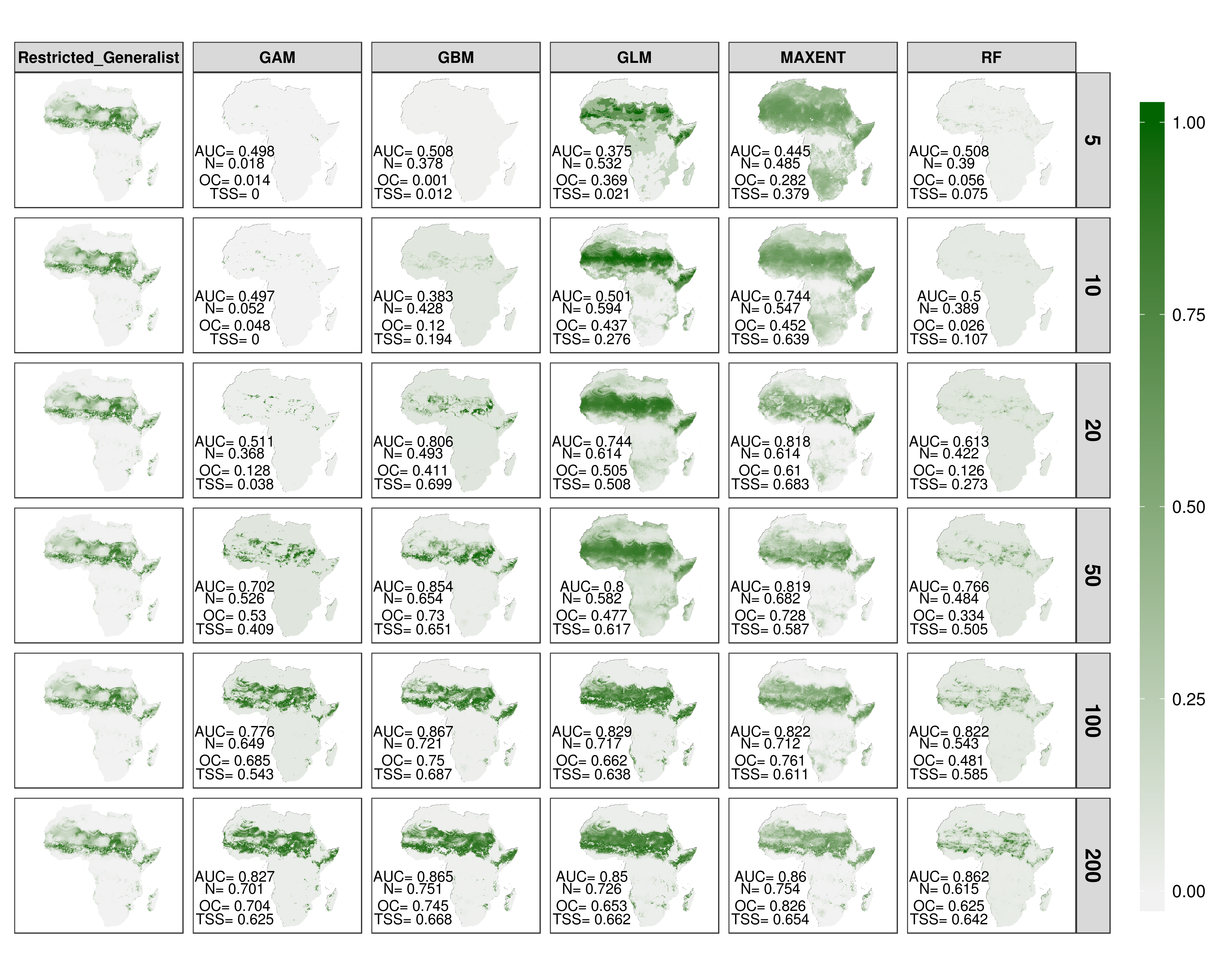


**Figure G: Spatial agreement between the predicted and “true” ranges for the restricted generalist species (low grid resolution).** The Y axis represents the number of species occurrences and the X axis is the model algorithms. Each plot has the value of AUC and TSS and the N stand for Niche overlap and OC stands for OCCC. The left column is the “true” ranges and the right columns are the predicted ones. The colour scale bar in the right side represents the suitability from 0 to 1.


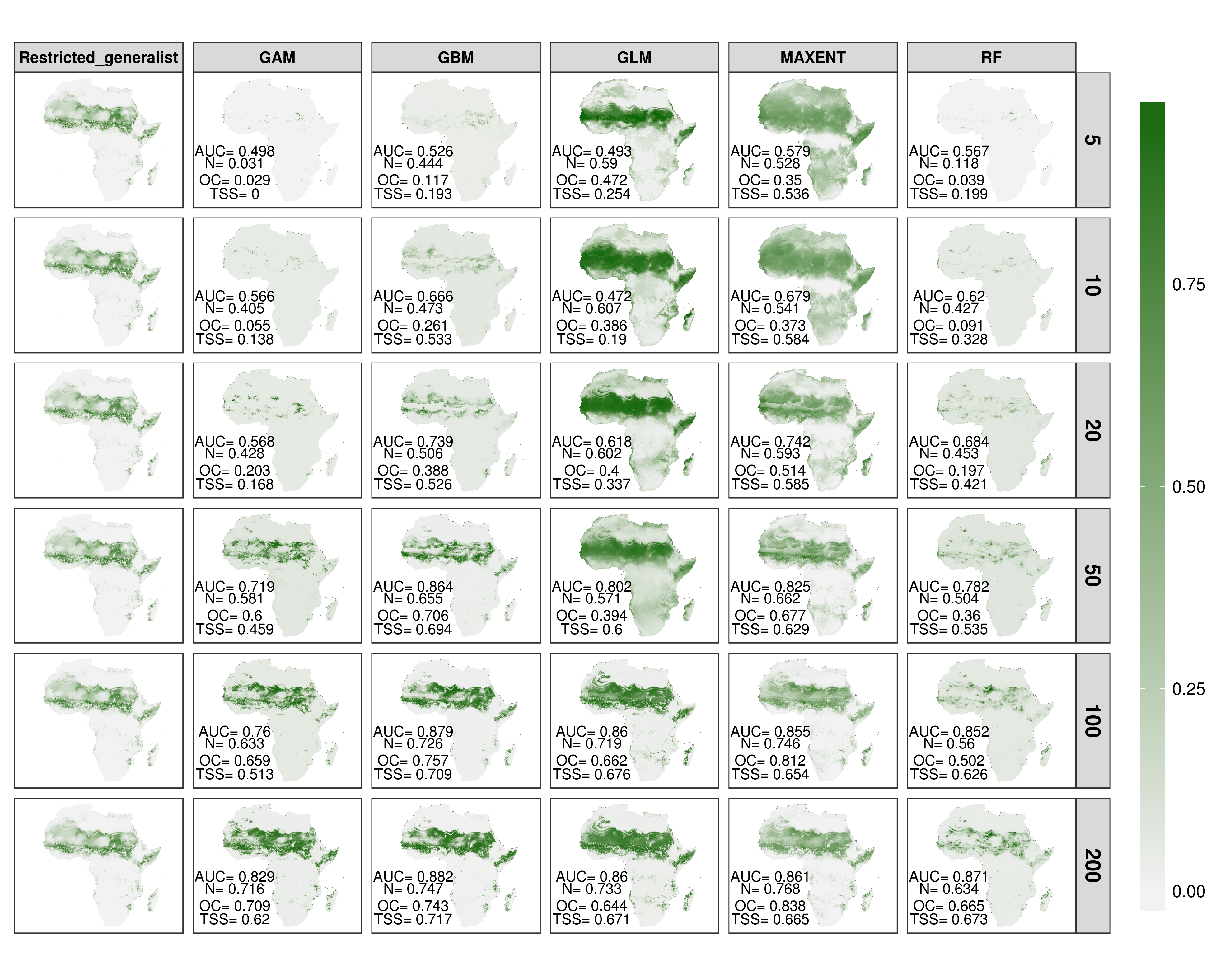
**Figure H: Spatial agreement between the predicted and “true” ranges for the restricted generalist species (high grid resolution).** The Y axis represents the number of species occurrences and the X axis is the model algorithms. Each plot has the value of AUC and TSS and the N stand for Niche overlap and OC stands for OCCC. The left column is the “true” ranges and the right columns are the predicted ones. The colour scale bar in the right side represents the suitability from 0 to 1.


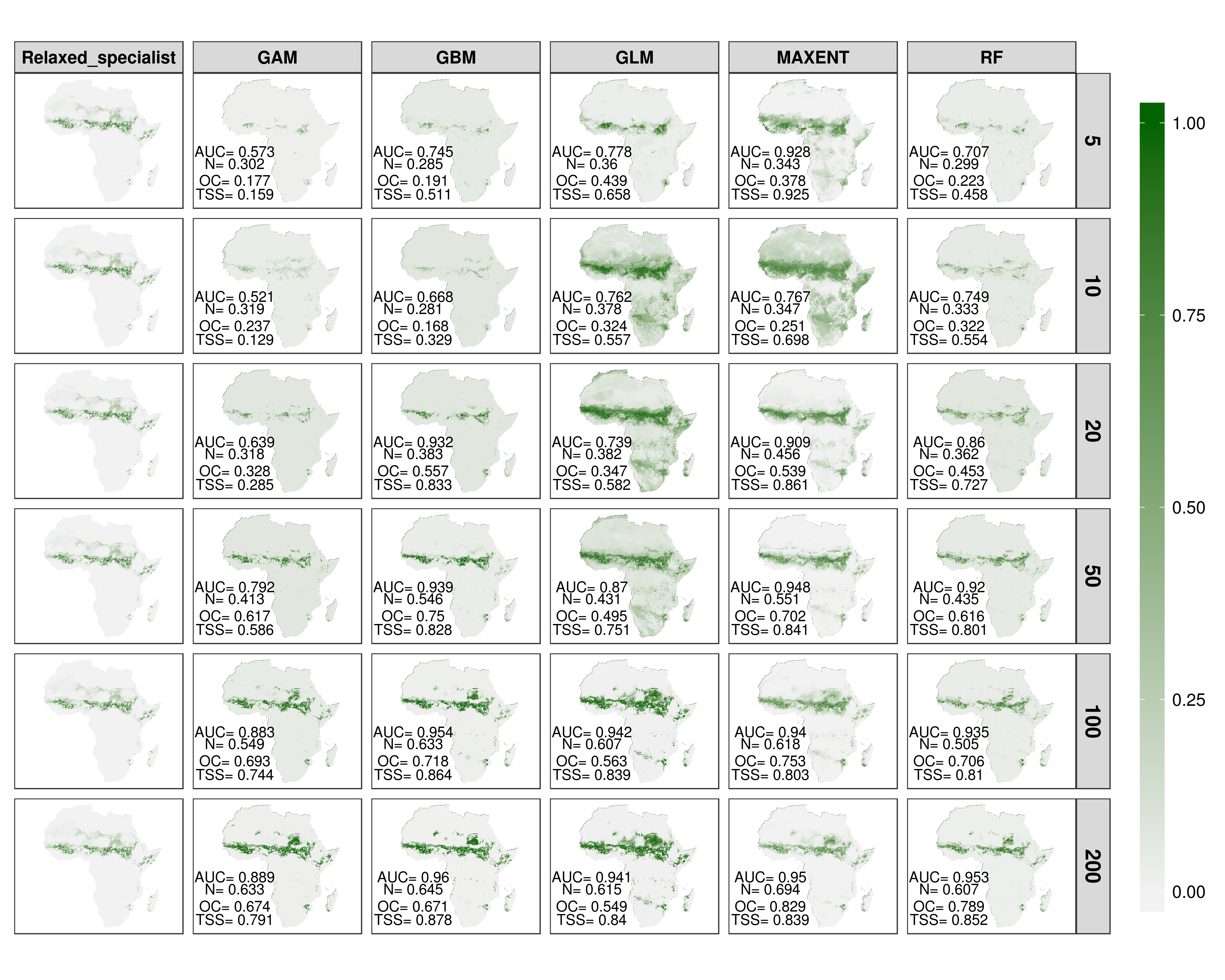
**Figure I: Spatial agreement between the predicted and “true” ranges for the relaxed specialist species (low grid resolution).** The Y axis represents the number of species occurrences and the X axis is the model algorithms. Each plot has the value of AUC and TSS and the N stand for Niche overlap and OC stands for OCCC. The left column is the “true” ranges and the right columns are the predicted ones. The colour scale bar in the right side represents the suitability from 0 to 1.


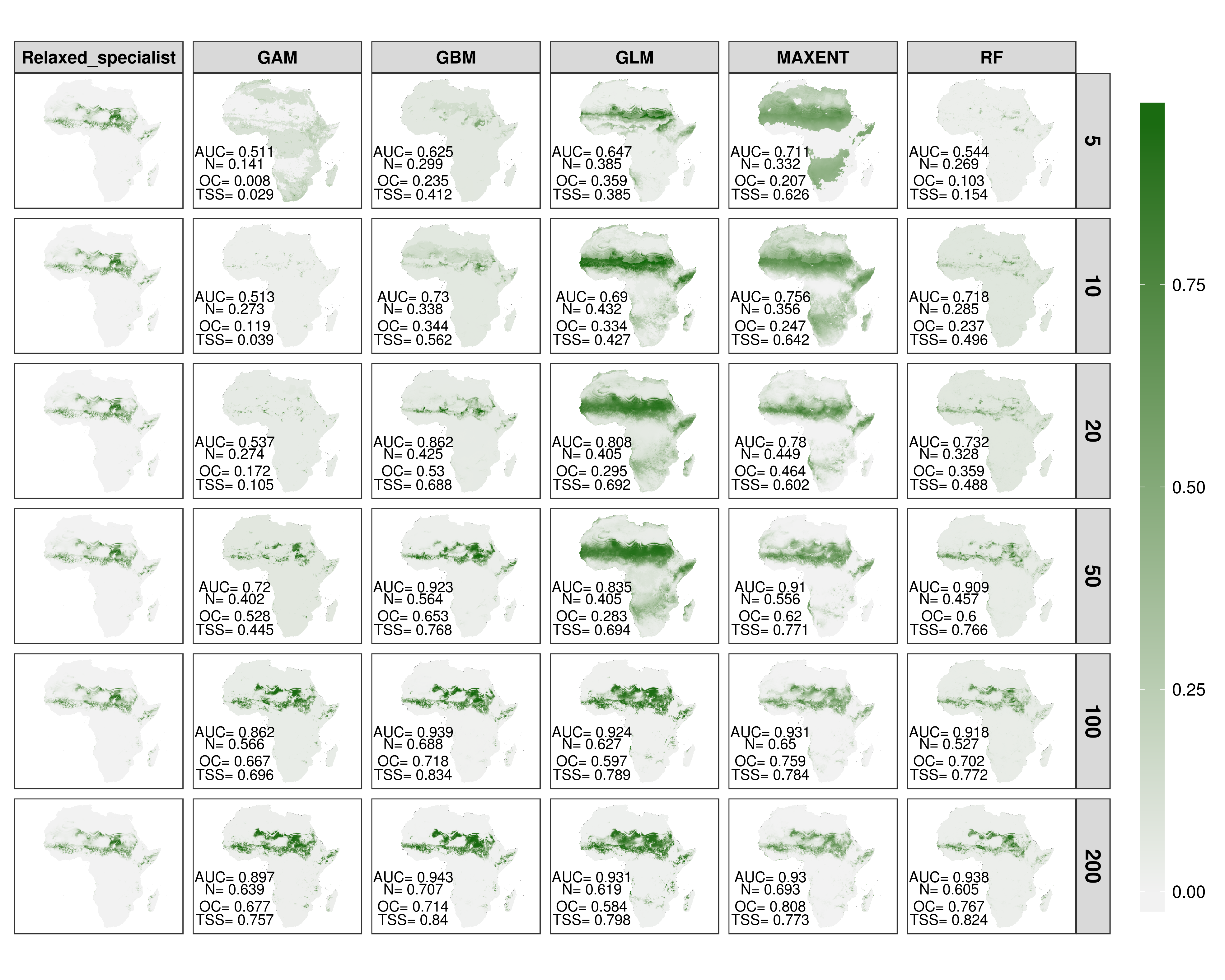
**Figure J: Spatial agreement between the predicted and “true” ranges for the relaxed specialist species (high grid resolution).** The Y axis represents the number of species occurrences and the X axis is the model algorithms. Each plot has the value of AUC and TSS and the N stand for Niche overlap and OC stands for OCCC. The left column is the “true” ranges and the right columns are the predicted ones. The colour scale bar in the right side represents the suitability from 0 to 1.


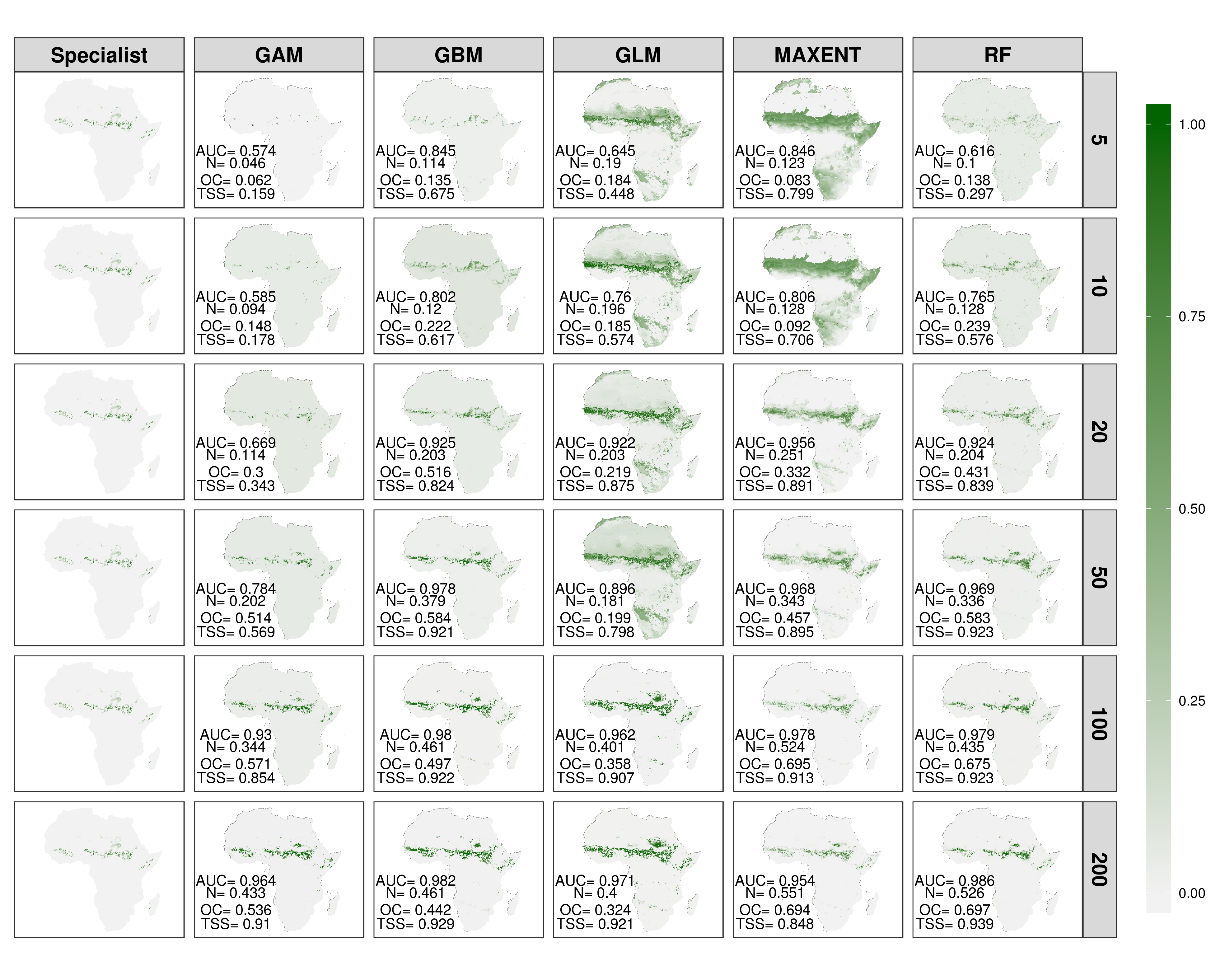
**Figure K: Spatial agreement between the predicted and “true” ranges for the specialist species (low grid resolution).** The Y axis represents the number of species occurrences and the X axis is the model algorithms. Each plot has the value of AUC and TSS and the N stand for Niche overlap and OC stands for OCCC. The left column is the “true” ranges and the right columns are the predicted ones. The colour scale bar in the right side represents the suitability from 0 to 1.


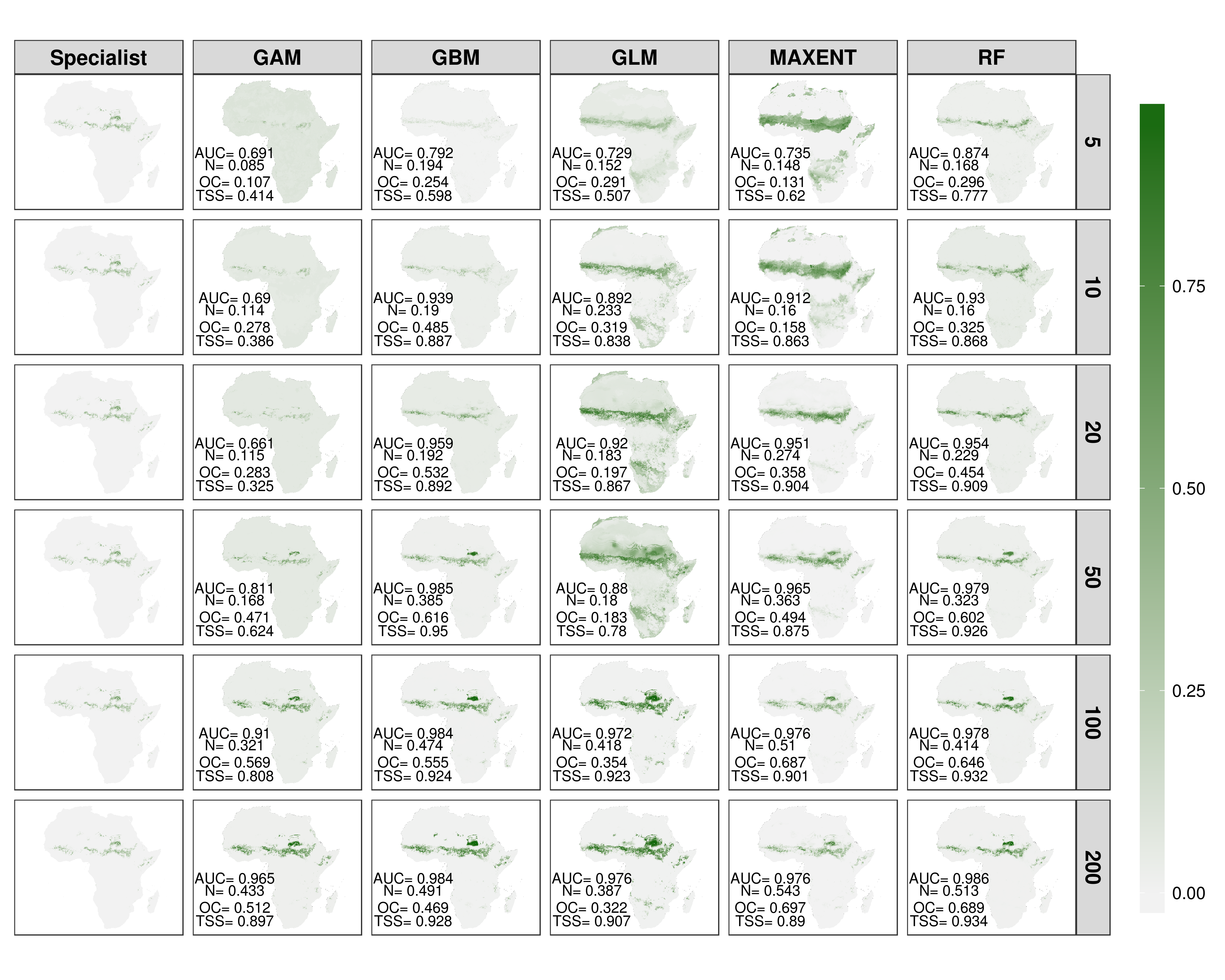
**Figure L: Spatial agreement between the predicted and “true” ranges for the specialist species (high grid resolution)**. The Y axis represents the number of species occurrences and the X axis is the model algorithms. Each plot has the value of AUC and TSS and the N stand for Niche overlap and OC stands for OCCC. The left column is the “true” ranges and the right columns are the predicted ones. The colour scale bar in the right side represents the suitability from 0 to 1.

**
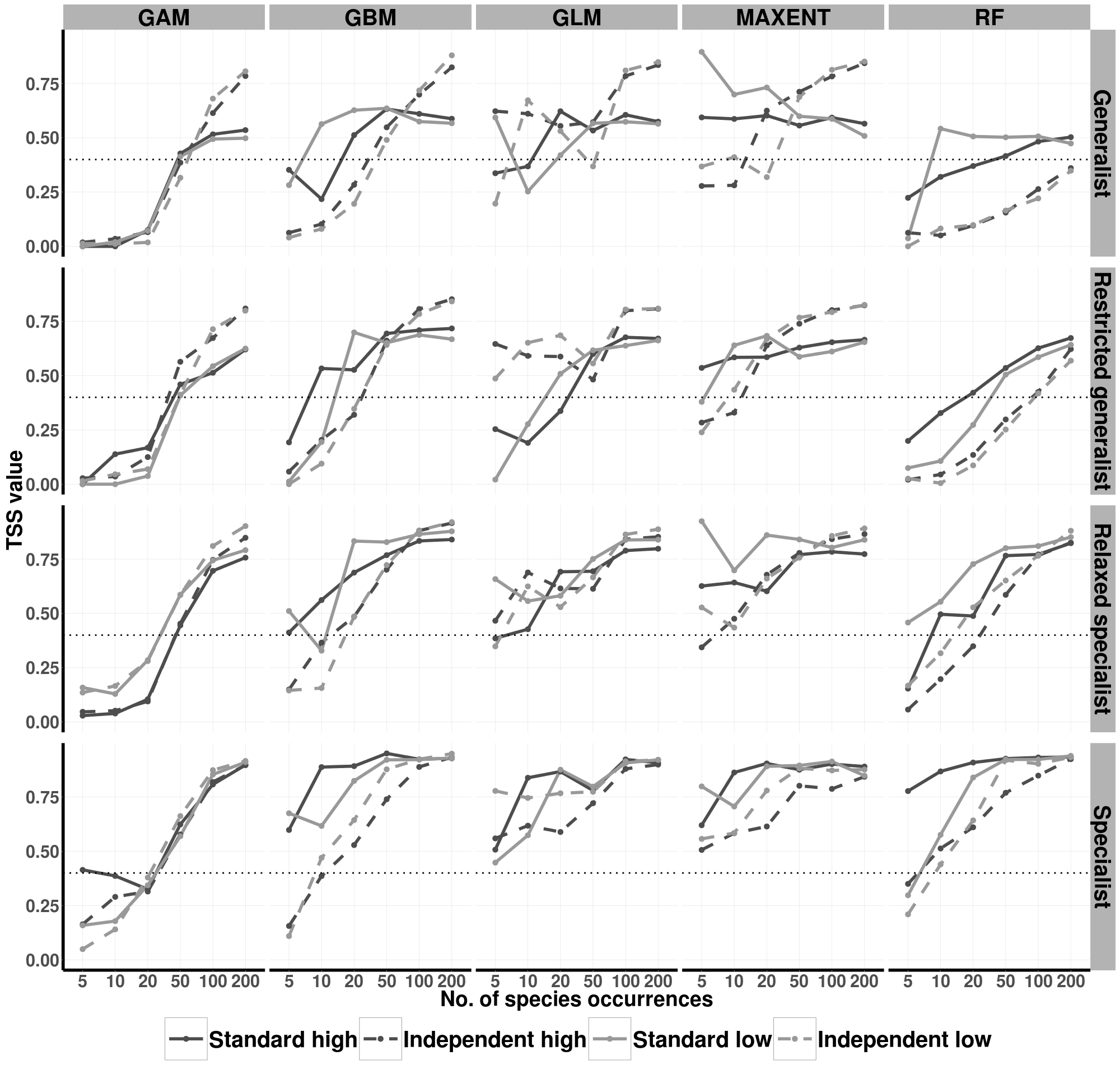
Figure M:** **The standard and independent TSS evaluation metrics values at low and high grid resolutions.** The solid lines represent the standard TSS and dashed lines represent the independent TSS, while the dark grey lines are high resolution and the light grey lines are low. The dotted line is the threshold value below which indicate poor model performance.


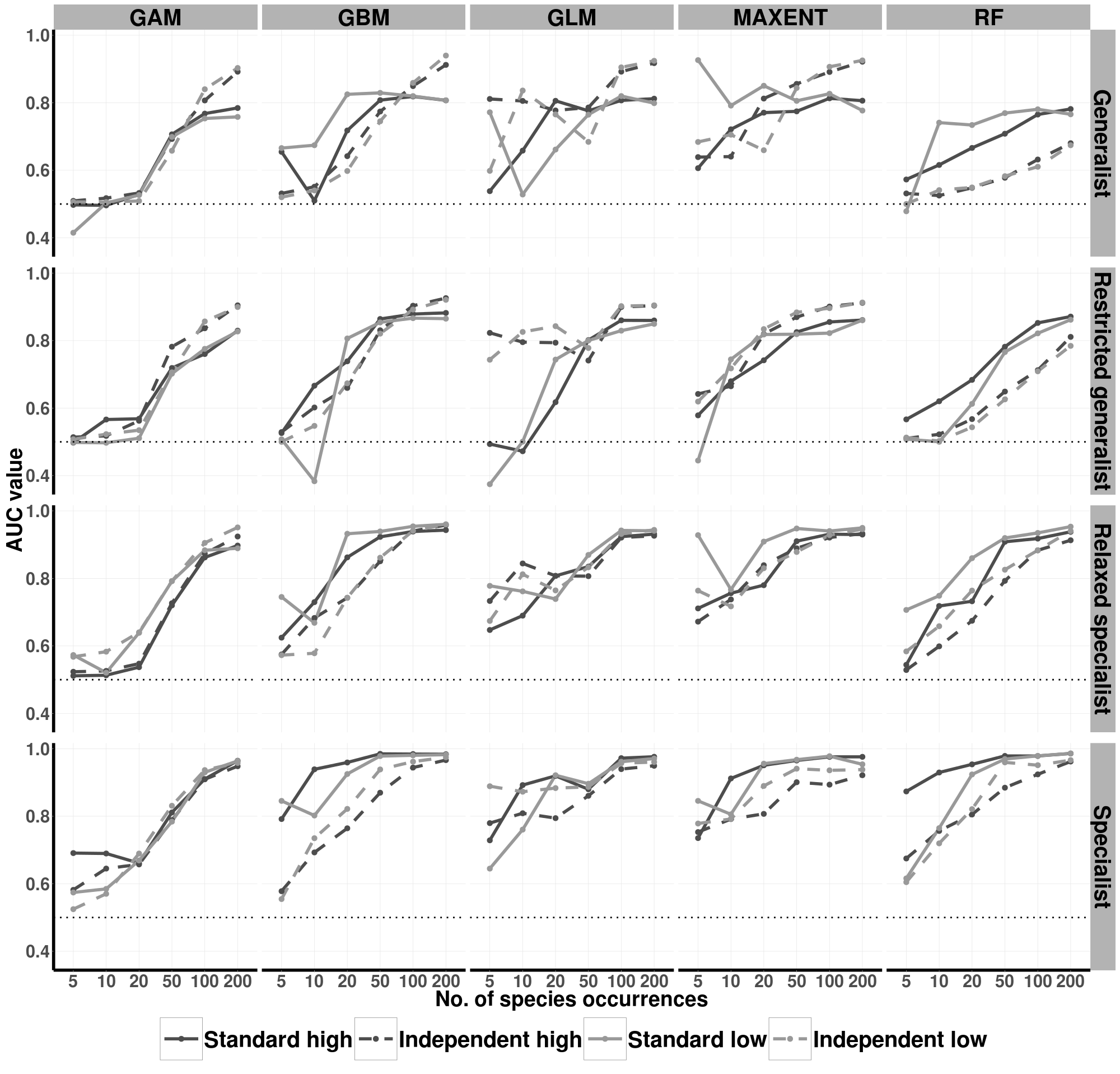
**Figure N:** **The standard and independent AUC evaluation metrics values at low and high grid resolutions.** The solid lines represent the standard AUC and dashed lines represent the independent AUC, while the dark grey lines are high resolution and the light grey lines are low. The dotted line is the threshold value below which indicate poor model performance.


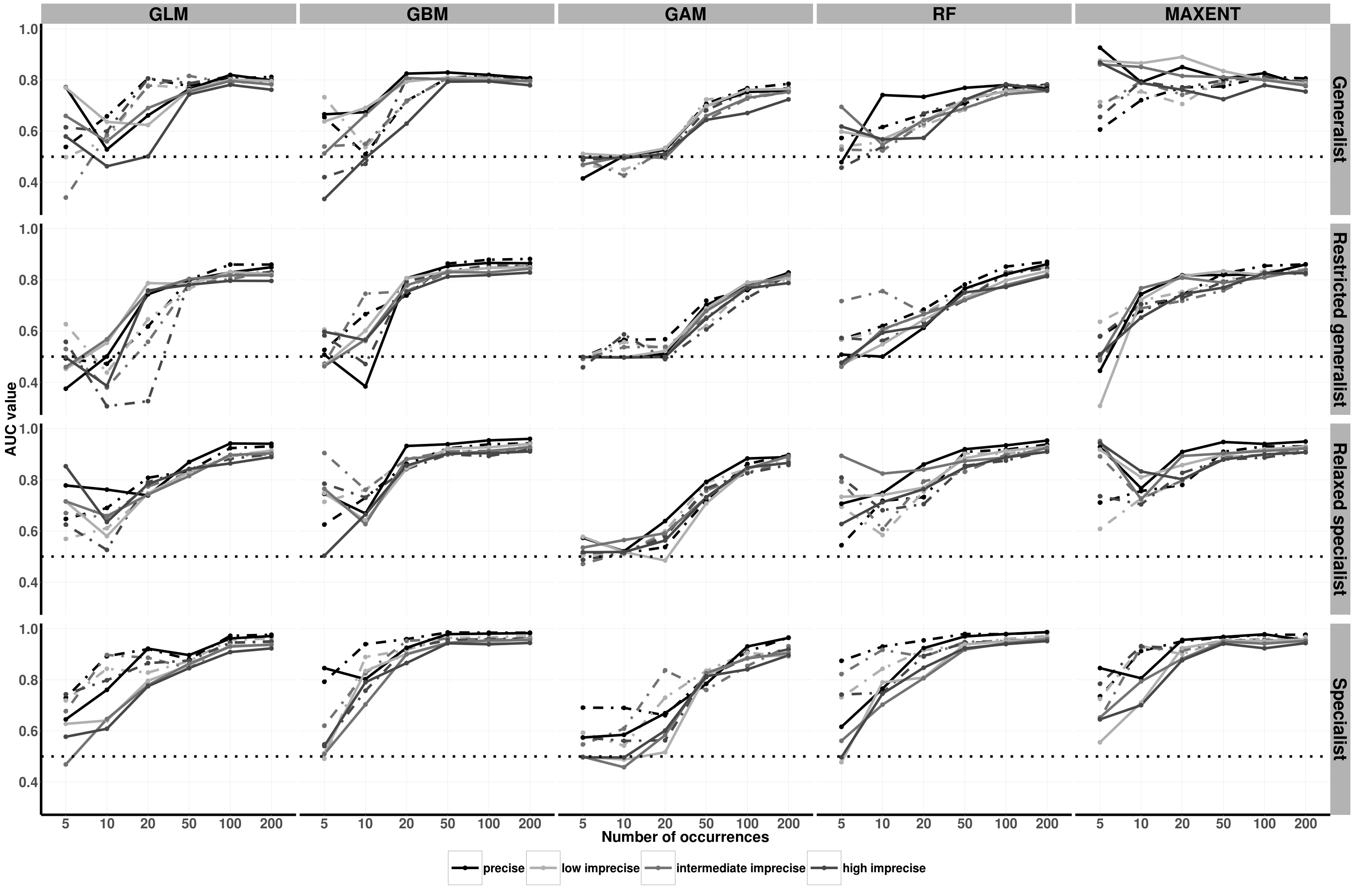
**Figure O: The standard AUC evaluation metrics for the model fitted with precise occurrences and imprecise occurrences.** The solid lines represent the low grid resolution, and the dashed lines, high resolution. The dotted line is the threshold value below which indicate poor model performance.


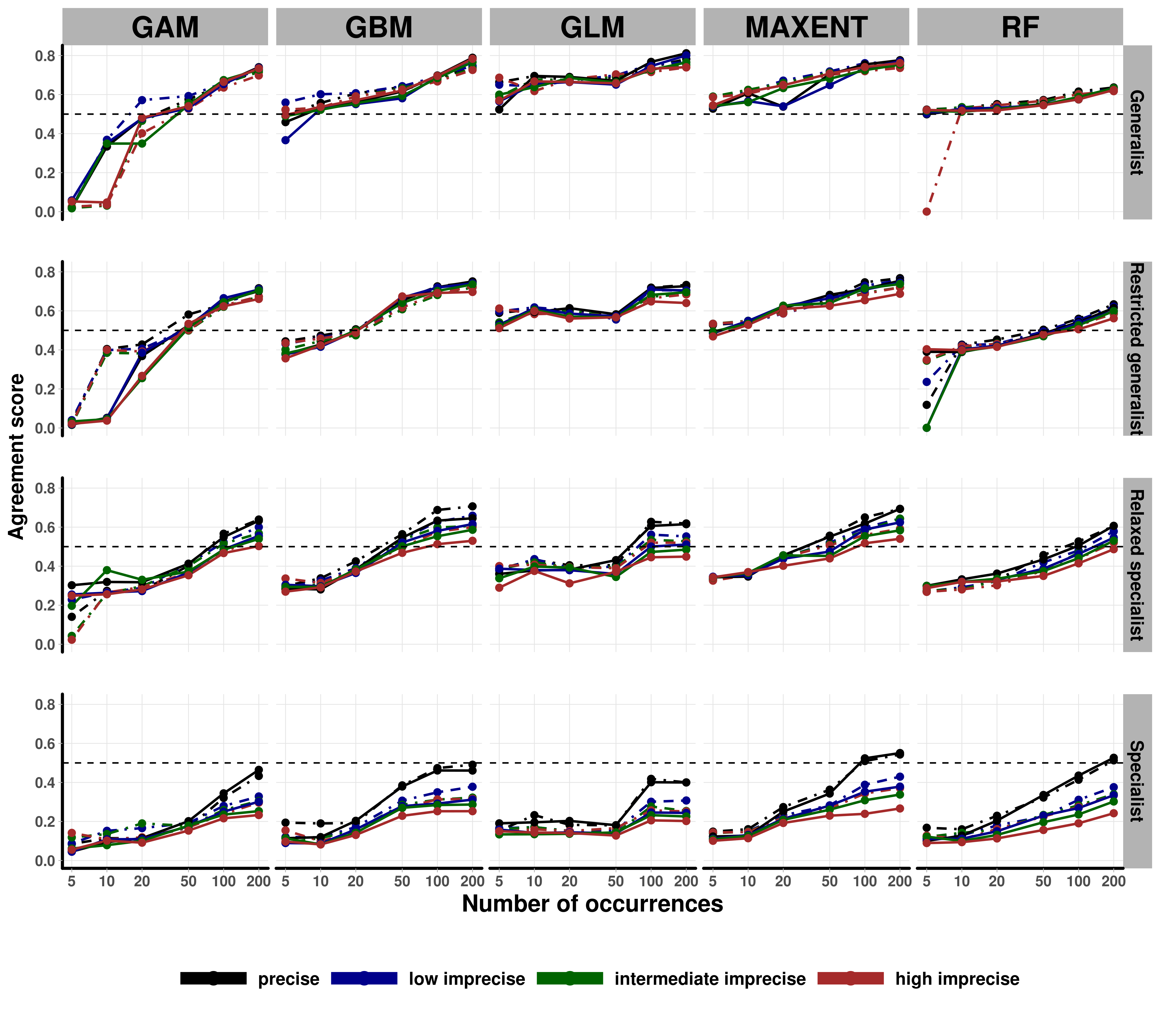
**Figure P:** **The agreement (according to niche-overlap index) between the predicted ranges with precise species' occurrences and the predicted ranges with imprecise species' occurrences for four different species at high and low grid resolutions**. The left y axis is scaled from 0 to 1, where 0 means no agreement and 1 is 100 % agreement. The solid lines represent the low grid resolution and dashed lines represent the high resolution. The line colour represents the precision levels of the species occurrences used.

**Table A:** **The list of the predictors remained after checking the collinearity by calculating the VIF and used to build the SDM**

Environmental variables Source Type

Bio 2—Annual Mean Diurnal Range

Bio 3—Isothermality

Bio 8—Mean Temperature of Wettest Quarter

Bio 9—Mean Temperature of Driest Quarter

Bio 13—Precipitation of Wettest Month

Bio 14—Precipitation of Driest Month [http://www.worldclim.org](http://www.worldclim.org/) Continuous

Bio 15—Precipitation Seasonality (CV)

Bio 18—Precipitation of Warmest Quarter

Bio 19—Precipitation of Coldest Quarter

Altitude

Roughness index

Land Cover [http://glcf.umd.edu](http://glcf.umd.edu/) Categorical

Net Primary production [http://neo.sci.gsfc.nasa.gov](http://neo.sci.gsfc.nasa.gov/) Continuous

Annual mean evapo-transpiration [http://earlywarning.usgs.gov](http://earlywarning.usgs.gov/) Continuous

Vegetation Continuous Field <http://glcf.umd.edu/data/vcf/> Continuous

**Table B: The details and settings of model implementation.**

**Table C**: **The standard and the independent AUC value for low grid resolution.** The column P refers to the p. value of the Wilcoxon test. The column standard AUC is the mean value of the AUC calculated during model evaluation. The column ind AUC refers to the AUC calculated using independent presence and true absence point. The column effect refers to the value of the effect size of the significance test. The column Diff is the differences between the standard and independent AUC

**Table D:** **The standard and the independent TSS value for low grid resolution.** The column P refers to the p. value of the Wilcoxon test. The column standard TSS is the mean value of the TSS calculated during model evaluation. The column ind TSS refers to the TSS calculated using independent presence and true absence point. The column effect refers to the value of the effect size of the significance test. The column Diff is the differences between the standard and independent TSS

**Table E:** **The standard and the independent AUC value for high grid resolution.** the column P refers to the p. value of the Wilcoxon test. The column standard AUC is the mean value of the AUC calculated during model evaluation. The column ind AUC refers to the AUC calculated using independent presence and true absence point. The column effect refers to the value of the effect size of the significance test. The column Diff is the differences between the standard and independent AUC

**Table F:** **The standard and the independent TSS value for high grid resolution.** The column P refers to the p. value of the Wilcoxon test. The column standard TSS is the mean value of the TSS calculated during model evaluation. The column ind TSS refers to the TSS calculated using independent presence and true absence point. The column effect refers to the value of the effect size of the significance test. The column Diff is the differences between the standard and independent TSS

**Table G:** **The significance of the Wilcoxon test between the AUC of the high and low resolutions.** The column P refers to the p. value of the Wilcoxon test. The column AUC high is the mean value of the AUC at high grid resolution. The column AUC low is the mean value of the AUC at low grid resolution. The column effect refers to the value of the effect size of the significance test. The column Diff stands for the differences between the AUC values at high and low grid resolutions

**Table H:** **The significance of the Wilcoxon test between the TSS of the high and low grid resolutions.** The column P refers to the p. value of the Wilcoxon test. The column TSS high is the mean value of the TSS at high grid resolution. The column TSS low is the mean value of the TSS at low grid resolution. The column effect refers to the value of the effect size of the significance test. The column Diff stands for the differences between the TSS values at high and low grid resolutions

**Table I: The regression coefficient quantified the effect of each level of each explanatory variables on the Species distibution performance**
