# Supplementary material for: The interplay of various sources of noise on reliability of species distribution models hinges on ecological specialisation
Source: PLoS One. 2017 Nov 13;12(11):e0187906. doi: 10.1371/journal.pone.0187906 (PMC5683637; doi:10.1371/journal.pone.0187906)
Supplement: S2 Appendix — (DOC) [file pone.0187906.s002.doc]

**S2 Appendix: Overall Concordance Correlation Coefficient**

Overall Concordance Correlation Coefficient (OCCC), is a measure of agreement between two continuous datasets which were generated using two different approaches [1]. This index is an expansion of the Concordance Correlation Coefficient introduced by Lin (1989), and is commonly used in clinical studies as a reliability index to assess the agreement between multiple diagnostic or therapeutic approaches [3]. This index calculates the precision and accuracy of the predicted data by measuring 1) the deviation of each predicted point from the fitted line, the line that minimizing the variance between the values of the “true” and the predicted ranges, and 2) the distance of the fitted line from the 45^◦^ isocline, and thus considers the variation between the two approaches [1,3]. We used the OCCC to compare spatially the “true” distribution range with the modelled ranges, and also to compare the models based on precise species occurrences with those based on imprecise. In this study, we computed the OCCC using the “epiR” R package [4]. The OCCC value varies between 0 and 1, with 0 representing 100% disagreement and 1 represents 100% agreement between the true and predicted ranges.

**REFERENCES**

1. Barnhart HX, Haber M, Song J. Overall Concordance Correlation Coefficient for Evaluating Agreement Among Multiple Observers. Biometrics. 2002;58: 1020–1027. doi:10.1111/j.0006-341X.2002.01020.x

2. Lin LI. A concordance correlation coefficient to evaluate reproducibility. Biometrics. 1989;45: 255–68. Available: http://www.ncbi.nlm.nih.gov/pubmed/2720055

3. King TS, Chinchilli VM. A generalized concordance correlation coefficient for continuous and categorical data. Stat Med. 2001;20: 2131–47. doi:10.1002/sim.845

4. Stevenson M, Nunes T, Heuer C, Marshall J, Sanchez J, Thornton R, et al. epiR: Tools for the

Analysis of Epidemiological Data. R package version 0.9-69.

Available:<https://cran.r-project.org/web/packages/epiR/index.html>. 2015.
