# Supplementary material for: The interplay of various sources of noise on reliability of species distribution models hinges on ecological specialisation
Source: PLoS One. 2017 Nov 13;12(11):e0187906. doi: 10.1371/journal.pone.0187906 (PMC5683637; doi:10.1371/journal.pone.0187906)
Supplement: S3 Appendix — (DOC) [file pone.0187906.s003.doc]

**S3 Appendix: The impact of the grid resolutions on SDMs outcomes.**

Our results revealed that grid resolution had no considerable effect on SDMs outcomes irrespective of species specialisation and model algorithm. Although there was a statistical difference between the high and low grid resolutions, it was relatively small (Tables S6-S7). Moreover, these differences were not consistent: in some cases, the high grid resolution resulted in higher performance than the low resolution, whereas in other cases the low grid resolution outperformed. These findings contrast with previous studies that concluded that using high grid resolution improves the predictive performance of SDMs [1,2]. However, our result is in line with Guisan *et al.* [3], who found an inconsiderable influence of grid resolution on SDMs outcomes. In our study, we observed the influence of grid resolution only when a low number of occurrences (≤ 20) were used (Fig 3 and S2-S4 Figs). We relate this influence to the prediction stability, where SDMs calibrated with low numbers of occurrences are likely to have a lower stability than those calibrated with higher numbers, which in turn might underlie the differences between high and low grid resolutions

**REFERENCES**

1. Austin MP, Van Niel KP. Improving species distribution models for climate change studies: variable selection and scale. J Biogeogr. 2011;38: 1–8. doi:10.1111/j.1365-2699.2010.02416.x

2. Rengstorf AM, Yesson C, Brown C, Grehan AJ. High-resolution habitat suitability modelling can improve conservation of vulnerable marine ecosystems in the deep sea. J Biogeogr. 2013;40: 1702–1714. doi:10.1111/jbi.12123

3. Guisan A, Graham CH, Elith J, Huettmann F. Sensitivity of predictive species distribution

models to change in grain size. Divers Distrib. 2007;13: 332–340.

doi:10.1111/j.1472-4642.2007.00342.x
